# Population Genomics of *emm4* Group A Streptococcus Reveals Progressive Replacement with a Hypervirulent Clone in North America

**DOI:** 10.1128/mSystems.00495-21

**Published:** 2021-08-10

**Authors:** Sruti DebRoy, Misu Sanson, Brittany Shah, Shirjana Regmi, Luis Alberto Vega, Chioma Odo, Pranoti Sahasrabhojane, Allison McGeer, Gregory J. Tyrrell, Nahuel Fittipaldi, Samuel A. Shelburne, Anthony R. Flores

**Affiliations:** a Department of Infectious Diseases Infection Control and Employee Health, MD Anderson Cancer Center, Houston, Texas, USA; b Division of Infectious Diseases, Department of Pediatrics, University of Texas Health Science Center, McGovern Medical School, Houston, Texas, USA; c Lunenfeld-Tanenbaum Research Institute, Sinai Health System, Toronto, Ontario, Canada; d Department of Laboratory Medicine and Pathobiology, University of Toronto, Toronto, Ontario, Canada; e Alberta Public Health Laboratories, Public Health–Alberta Health Services, Edmonton, Alberta, Canada; f Division of Diagnostic and Applied Microbiology, Department of Laboratory Medicine and Pathology, University of Alberta, Edmonton, Alberta, Canada; g Public Health Ontario Laboratory, Toronto, Ontario, Canada; h Department of Genomic Medicine, MD Anderson Cancer Center, Houston, Texas, USA; i Center for Antimicrobial Resistance and Microbial Genomics, University of Texas Health Science Center McGovern Medical School, Houston, Texas, USA; Vanderbilt University

**Keywords:** M type, evolution, exotoxin, genomics, group A *Streptococcus*, population genetics, virulence

## Abstract

Clonal replacement is a major driver for changes in bacterial disease epidemiology. Recently, it has been proposed that episodic emergence of novel, hypervirulent clones of group A Streptococcus (GAS) results from acquisition of a 36-kb DNA region leading to increased expression of the cytotoxins Nga (NADase) and SLO (streptolysin O). We previously described a gene fusion event involving the gene encoding the GAS M protein (*emm*) and an adjacent M-like protein (*enn*) in the *emm4* GAS population, a GAS *emm* type that lacks the hyaluronic acid capsule. Using whole-genome sequencing of a temporally and geographically diverse set of 1,126 isolates, we discovered that the North American *emm4* GAS population has undergone clonal replacement with emergent GAS strains completely replacing historical isolates by 2017. Emergent *emm4* GAS strains contained a handful of small genetic variations, including the *emm-enn* gene fusion, and showed a marked *in vitro* growth defect compared to historical strains. In contrast to other previously described GAS clonal replacement events, emergent *emm4* GAS strains were not defined by acquisition of exogenous DNA and had no significant increase in transcript levels of *nga* and *slo* toxin genes via RNA sequencing and quantitative real-time PCR analysis relative to historic strains. Despite the *in vitro* growth differences, emergent *emm4* GAS strains were hypervirulent in mice and *ex vivo* growth in human blood compared to historical strains. Thus, these data detail the emergence and dissemination of a hypervirulent acapsular GAS clone defined by small, endogenous genetic variation, thereby defining a novel model for GAS strain replacement.

**IMPORTANCE** Severe invasive infections caused by group A Streptococcus (GAS) result in substantial morbidity and mortality in children and adults worldwide. Previously, GAS clonal strain replacement has been attributed to acquisition of exogenous DNA leading to novel virulence gene acquisition or increased virulence gene expression. Our study of type *emm4* GAS identified emergence of a hypervirulent GAS clade defined by variation in endogenous DNA content and lacking augmented toxin gene expression relative to replaced strains. These findings expand our understanding of the molecular mechanisms underlying bacterial clonal emergence.

## INTRODUCTION

Bacterial clonal emergence is a hallmark of human epidemics and has contributed to changes in disease caused by Staphylococcus aureus (1, 2), Streptococcus pneumoniae (3), and Neisseria meningitidis (4) to name a few. Streptococcus pyogenes (group A Streptococcus [GAS])—a leading cause of invasive bacterial disease in humans—has long served as a model organism for investigating severe bacterial infections and bacterial epidemic behavior (5–7). The primary typing scheme for GAS is based on sequencing of the hypervariable 5′ end of the *emm* gene which encodes the cell surface M protein, with GAS strains of the same *emm* type generally being more closely related to each other than to strains of different *emm* types (8). GAS epidemiology is characterized by epidemic events resulting from strain emergence and eventual replacement of prior circulating strains. To date, emergence of GAS clones has primarily been attributed to acquisition of exogenous DNA (6, 9). For example, the replacement of historical *emm1* strains with the currently circulating pandemic *emm1* GAS in the 1980s resulted from recombination of a 36-kb region that contained the secreted NADase (encoded by *nga*) and streptolysin O (SLO) (*slo*) toxins, two well-known, cotranscribed virulence factors (10–13). This recombination event resulted in a high-activity *nga* or *slo* promoter which has been linked to increased frequency and severity of infections and rapid global spread (6, 14). A similar recombination event has since been described in *emm89* GAS strains contributing to the emergence of a new and more virulent clade (7). In addition to increased Nga/SLO expression, contemporary *emm1* GAS acquired via horizontal gene transfer (HGT) a novel prophage-associated pyrogenic exotoxin (SpeA), further contributing to virulence potential and spread (6)—a mechanism also attributed to the enhanced invasiveness of *emm3* GAS strains following integration of a bacteriophage carrying a novel phospholipase (Sla) (15).

The GAS hyaluronic acid capsule has historically been considered critical to virulence by promoting resistance to host immune cell phagocytosis (16–20). However, several *emm* types that lack capsule have recently been identified (21, 22). There has been a significant rise in the number of infections caused by acapsular strains (9, 22), and they now collectively account for nearly one-third of invasive GAS infections (23, 24). Interestingly, lack of capsule production through gene loss or mutation has been linked to high-activity *nga* and *slo* genotypes (25). The combination was first described in *emm89* GAS strains (7, 26) but has since been recognized to occur in additional GAS genotypes, including *emm28* and *emm87* (25). Thus, the current paradigm is that acapsular GAS bacteria compensate for the lack of capsule by elevating Nga/Slo production.

We recently reported the occurrence of a chimeric *emm* gene in circulating acapsular *emm4* GAS strains isolated in the United States and England (27). The new *emm4* strains harboring the chimeric *emm* gene completely replaced the circulating strains by 2017 in the Houston, Texas, metropolitan area (27). To better understand the timeline and the genetic changes driving the emergence of chimeric *emm4* strains, we analyzed 1,126 temporally and geographically diverse *emm4* strains from the United States, Canada, and United Kingdom. Our analysis reveals that a new, genetically distinct clade of *emm4* GAS strains with enhanced virulence has emerged and constitutes nearly the entire contemporary *emm4* population. In striking contrast to previous GAS clonal replacement events, we report here that the newly circulating *emm4* strains are defined by small genetic changes of existing DNA rather than acquisition of exogenous DNA and do not have augmented Nga/SLO production.

## RESULTS

### Analysis of the *emm4* GAS population structure reveals distinct clades with nearly complete temporal replacement.

**FIG 1:**
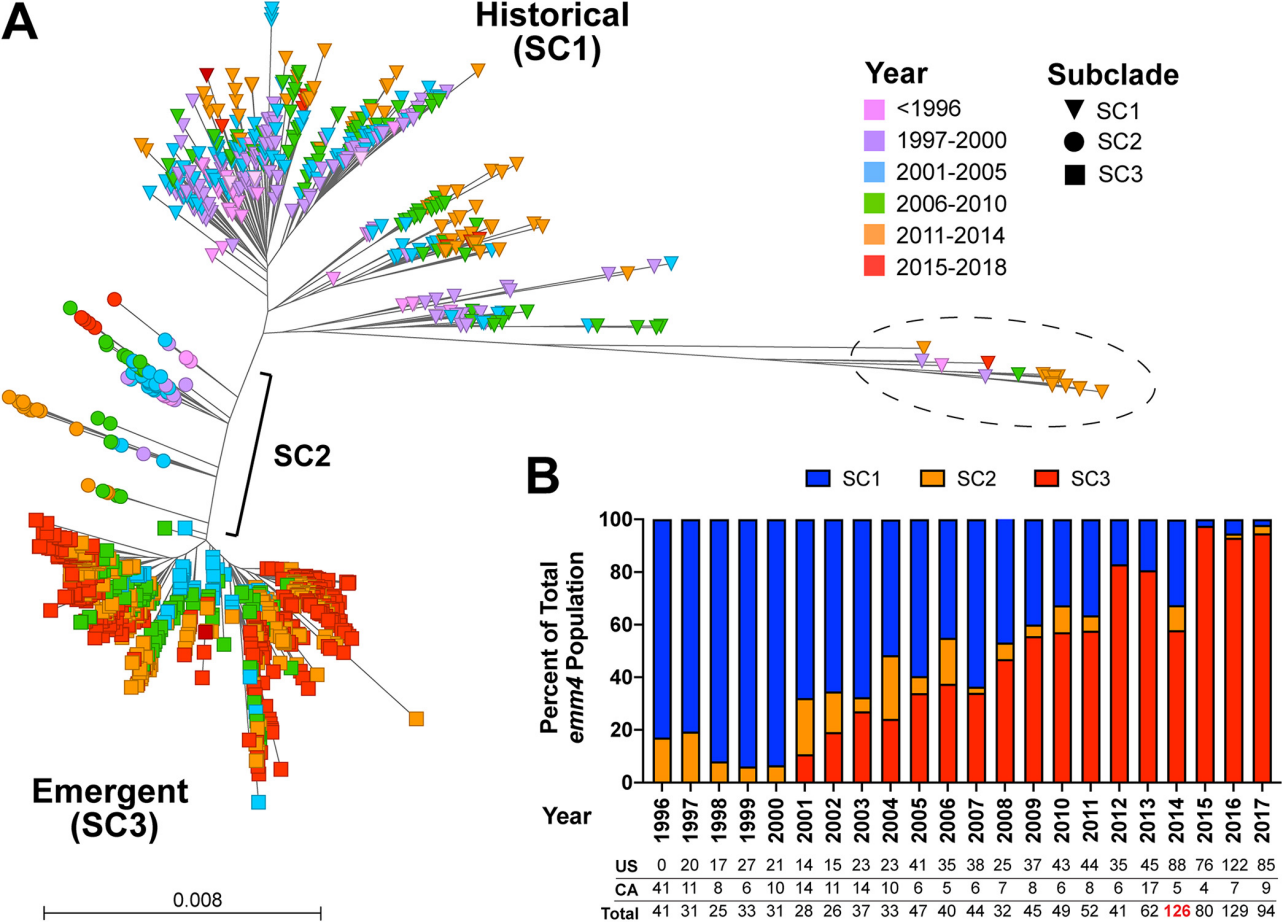
Maximum likelihood phylogenetic reconstruction of 1,126 *emm4* GAS disease isolates from North America and the United Kingdom. (A) Unrooted neighbor-joining phylogenetic tree based on 12,436 biallelic SNPs relative to the *emm4* reference strain Duke (GenBank accession number CP031770) following correction for recombination (ClonalFrameML). Branch tips colored to indicate individual *emm4* strain year of isolation and shape for subclade designation (BAPS). The dashed circle indicates single-locus variant (ST38) strains from the main (ST39) *emm4* population. (B) Progressive replacement of historical (SC1) strains by emergent (SC3) over the 22-year period. The total number of isolates and geographic origin are indicated below bar graph. The red value for 2014 indicates that 32 isolates from the United Kingdom were included in the analysis.

A total of 861 strains were sequenced for the current study, and we analyzed the genome sequences from an additional 265 (*n* = 1,126 total sequences) *emm4* GAS disease isolates (invasive and noninvasive) originating from multiple geographic locations in the United States, Canada, and United Kingdom, spanning 25 years (Table 1; see also Table S1 in the supplemental material). Importantly, >70% of the isolates were derived from national-level surveillance (Centers for Disease Control and Prevention, Active Bacterial Core Surveillance program) spanning nearly 20 years (Table 1). Mean sequencing depth of coverage exceeded 120-fold for all strains analyzed. Phylogenetic relationships were inferred based on single nucleotide polymorphisms (SNPs) relative to the core genome (excluding mobile genetic elements [MGE]) of the reference *emm4* GAS strain Duke (GenBank accession number CP031770) (28). Based on Bayesian population clustering, the *emm4* GAS population consisted of three distinct subpopulations (subclades [SC]) (29) (Fig. 1A). Subclade 3 (SC3) was the most common subclade representing 54.5% (614/1,126) of *emm4* strains followed by SC1 (38.4% [433/1,126]) and SC2 (7.0% [79/1,126]). SC1 included 16 isolates (dashed circle in Fig. 1A) that were a single locus variant (ST38) of the major *emm4* sequence type (ST39).

**TABLE 1:** Description of strains used in this study

Country	State/region	Years	No. of strains	Disease type	Reference(s)
Canada	Multiple^*a*^	1993−2018	221	Invasive	This study
United Kingdom	NA^*b*^	2014	33	Invasive/scarlet fever	76
United States	Multiple^*c*^	1998−2017	799	Invasive	23, 24^*d*^; this study
United States	Texas	2014−2017	74	Invasive/pharyngeal	22; this study

a Strains selected from a total of 884 strains with representatives from all 10 Canadian provinces.

b NA, not available.

c Total of 10 ABCs collection sites (https://www.cdc.gov/abcs/index.html).

d All GAS isolates beginning in 2015 were sequenced by the Centers for Disease Control and Prevention Streptococcal Laboratory with data publicly available at NCBI (BioProject accession number PRJNA395240).

10.1128/mSystems.00495-21.1 TABLE S1 Strains used in this study. Download Table S1, XLSX file, 0.10 MB. Copyright © 2021 DebRoy et al. 2021 DebRoy et al. https://creativecommons.org/licenses/by/4.0/ This content is distributed under the terms of the Creative Commons Attribution 4.0 International license.

Maximum likelihood phylogeny suggested that contemporary strains are overrepresented in SC3 compared to SC1 (note the predominance of red for SC3 strains in Fig. 1A). Thus, we determined the overall temporal distribution of the *emm4* GAS population stratified by Bayesian clustering (29). Consistent with clonal replacement, we observed a progressive decrease in SC1 strains and a concomitant increase in SC3 over time such that by 2017, 94.7% (89/94) of *emm4* strains sampled were SC3 (Fig. 1B). Interestingly, SC2 strains appear sporadically and do not persist over time. Thus, we chose to focus our analyses on the differences between SC1 and SC3 subclades. To better determine the timing of SC3 emergence, we next performed Bayesian reconstruction of ancestral states using BactDating (30). The overall substitution rate (3.5 substitutions per core genome per year; 1.84 × 10^−6^ substitutions per site year^−1^) was similar to that reported for *emm1* and *emm12* GAS using BEAST (31). The root date estimate (time to most recent common ancestor) for the entire *emm4* population was 1,921 (range, 1,886 to 1,936), and the date of emergence for SC2 strains was 1,979 (range, 1,975 to 1,983) compared to 1,992 (range, 1,991 to 1,993) for SC3 strains (see Fig. S1 in the supplemental material). For comparison, estimated date of SC1 emergence (excluding ST38 strains) was 1,955 (range, 1,946 to 1,964). Overall, these data indicate a strong temporal signal within the *emm4* population examined. Thus, henceforth, we refer to the SC1 strains as “historical” and to SC3 strains as “emergent.”

10.1128/mSystems.00495-21.7 FIG S1 Rooted phylogram of 1,126 *emm4* GAS strains showing subclade temporal relationships. Phylogram corrected for recombination using ClonalFrameML. Tree tips are colored by subclade (SC) as indicated in the legend. BactDating was used to infer node dates. Estimated dates for the most recent common ancestor (Root) and SC emergence are indicated. Download FIG S1, JPG file, 0.4 MB. Copyright © 2021 DebRoy et al. 2021 DebRoy et al. https://creativecommons.org/licenses/by/4.0/ This content is distributed under the terms of the Creative Commons Attribution 4.0 International license.

### Major gene content differences between clades are defined by prophage gene loss but do not involve MGE-related exotoxins or DNases.

**FIG 2:**
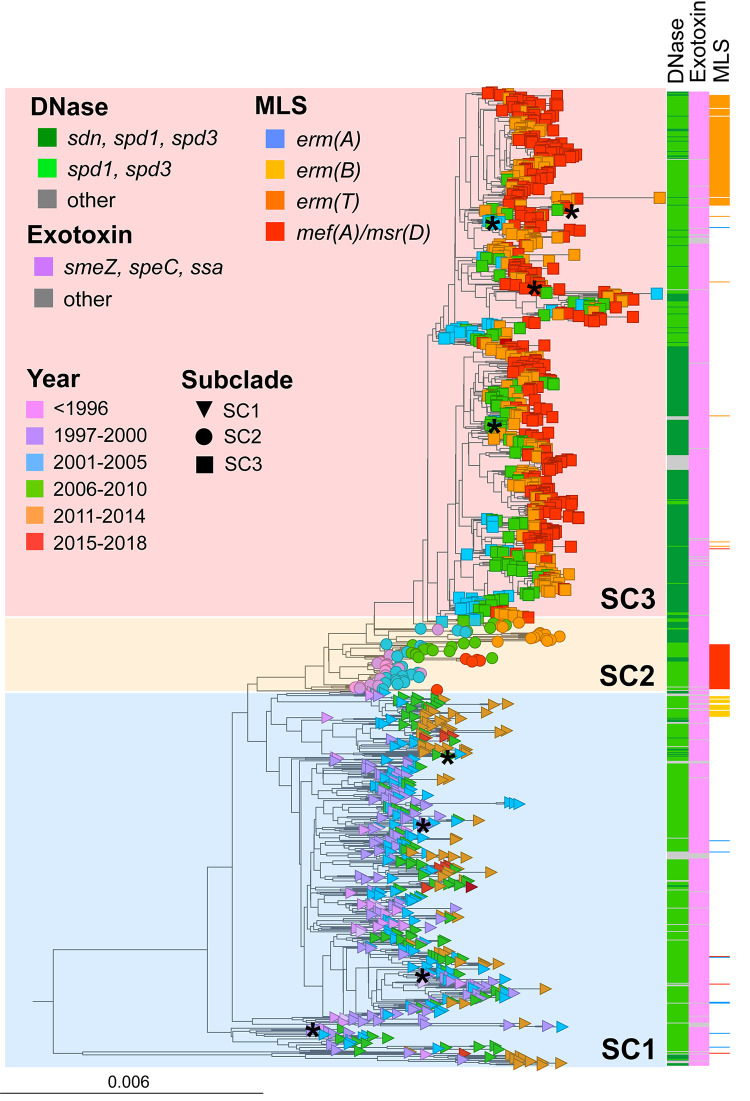
Distribution of DNases, exotoxins, and macrolide-lincosamide-streptogramin (MLS) resistance within the *emm4* population. A rooted maximum likelihood (ML) phylogram of 1,126 *emm4* GAS disease strains is shown. Branch tip color and shape indicate year and subclade designation as in Fig. 1 and the associated legend. Subclades (SC1 to 3) are indicated by shaded boxes. DNase, exotoxin, and antimicrobial resistance gene content is indicated by vertical bars to the right of the ML phylogram and defined in the legend headings. Asterisks indicate nodes representing strains with completed genomes (see Table S3 in the supplemental material).

Recently, it has been shown that contemporary *emm4* lineages are defined by prophage-specific gene loss resulting in cryptic prophages (32). Importantly, prophage degradation in the *emm4* lineages did not alter exotoxin or DNase gene content. Thus, we first compared the prophage-associated exotoxin and DNase gene content in our *emm4* population to determine differences between subclades. Among the major subclades, we observed no differences in exotoxin gene (*smeZ*, *speC*, and *ssa*) content (Fig. 2 and Table S2). Similarly, the prophage-associated, DNase-encoding genes *spd1* and *spd3* were nearly universally present in all three subclades (Fig. 2 and Table S2). However, the *emm4* subpopulations differed in the presence of the prophage-associated DNase gene *sdn* occurring in 54% (333/614) of SC3 strains but was not a subclade-defining feature (Fig. 2 and Fig. S2).

10.1128/mSystems.00495-21.2 TABLE S2 Exotoxin and DNase content of *emm4* GAS population. Download Table S2, DOCX file, 0.02 MB. Copyright © 2021 DebRoy et al. 2021 DebRoy et al. https://creativecommons.org/licenses/by/4.0/ This content is distributed under the terms of the Creative Commons Attribution 4.0 International license.

10.1128/mSystems.00495-21.8 FIG S2 Pangenome analysis and clustering of *emm4* GAS population using PANINI. Points represent individual strain clusters defined by the PANINI output and visualized using Microreact (microreact.org). Points are colored by subclade as defined in the legend. The dashed lines separate clusters based on gene content [*sdn*, *erm*(B), and *mef*(A)*/msr*(D)]. The boxed inset represents the unrooted neighbor-joining tree as in Fig. 1A with branch tips colored by subclade. Download FIG S2, JPG file, 0.2 MB. Copyright © 2021 DebRoy et al. 2021 DebRoy et al. https://creativecommons.org/licenses/by/4.0/ This content is distributed under the terms of the Creative Commons Attribution 4.0 International license.

10.1128/mSystems.00495-21.3 TABLE S3 Completed *emm4* GAS genomes used for transcriptome analysis. Download Table S3, DOCX file, 0.02 MB. Copyright © 2021 DebRoy et al. 2021 DebRoy et al. https://creativecommons.org/licenses/by/4.0/ This content is distributed under the terms of the Creative Commons Attribution 4.0 International license.

We next performed a pangenome analysis to further define gene content differences within the *emm4* population. The core genome (shared by >99% of strains) was defined by 1,516 (31.7%) of the total 4,788 genes identified in the *emm4* pangenome. In comparison, there are 1,934 genes in the Duke reference genome. The majority (2,879/4,788 [60.1%]) of genes were found in <15% of strains. Using a recently described, novel machine-learning method (PANINI) (33), we discovered that gene content differences contributing to *emm4* GAS population structure correlated almost perfectly with subclades defined by Bayesian population clustering from SNPs (Fig. S2)—an observation potentially consistent with previously described prophage degradation (32). In addition, genes conferring resistance to macrolides [e.g., *mef*(A) and *erm*(B)] also contributed to strain subclustering (Fig. S2). Interestingly, a subpopulation of SC3 strains contained *erm*(T) residing on a previously described ∼5-kb plasmid (34) (Fig. 2). However, strains were more broadly distributed among the SC3 population when assessing gene content (Fig. S2) compared to other resistance elements (data not shown). Further, subpopulation structure within SC3 strains was strongly associated with the presence of the DNase *sdn* (Fig. S2). Taken together, these data demonstrate that acquisition of an exogenous virulence factor does not distinguish emergent from historical *emm4* strains.

### Emergent clade *emm4* GAS strains are defined by small genetic changes and not large-scale recombination.

Previous studies of GAS epidemic clone emergence established that large recombination events, specifically involving the known GAS virulence factors Nga and SLO, are critical for epidemic clone emergence (6, 7). Examination of phylogenetic relationships before and after correction for recombination (ClonalFrameML and gubbins) (35, 36) revealed only modest changes in the *emm4* GAS population structure (Fig. S3A to C). To further facilitate identification of recombination, we sequenced to closure the genomes of eight (four SC1 and four SC3) *emm4* GAS strains representing major clades/subclades and MGE content (asterisks in Fig. 2 and Table S3). Given the sporadic temporal appearance and lack of persistence in SC2 strains, we focused our analyses on differences between SC1 and SC3. Comparison of the completed genomes revealed no large-scale (e.g., >1-kb) recombination, and *nga* and *slo*, including the associated promoter, were identical between completed genomes and within the studied *emm4* population (data not shown). Specifically, all strains possessed an identical “high-activity” *nga* or *slo* promoter (25). For comparison, in a study of *emm89* GAS clonal emergence, HGT and recombination accounted for the majority of core chromosomal differences between clades and ranged in size from a few hundred bases to >70 kb (26). Thus, although previous studies identified HGT as a hallmark of GAS clade emergence, these data show that HGT is not a major contributor to clade differences in the *emm4* GAS population.

10.1128/mSystems.00495-21.9 FIG S3 Exogenous DNA recombination and/or acquisition do not play a major role in the *emm4* GAS population. (A and B) Unrooted neighbor-joining phylogenetic tree inferred from 12,436 biallelic SNP loci prior to (A) and after correction for recombination using ClonalFrameML (B). Tree tips are colored to indicate year and shape to indicate subclade (SC) designation as defined in the legend. (C) Map of recombination relative to the reference genome Duke following gubbins and visualized using phandango (https://jameshadfield.github.io/phandango/). Regions of recombination are limited to MGE (e.g. prophage) and small genomic loci (e.g., *emm-enn* gene fusion). Historical clade strains are highlighted in blue. Download FIG S3, JPG file, 1.5 MB. Copyright © 2021 DebRoy et al. 2021 DebRoy et al. https://creativecommons.org/licenses/by/4.0/ This content is distributed under the terms of the Creative Commons Attribution 4.0 International license.

We next determined differences between emergent and historical strains with respect to small genetic changes (e.g., SNPs, insertions/deletions). In addition to the previously described *emm*-*enn* gene fusion (27), emergent strains were defined by a small number of differences in core chromosomal loci compared to historical strains. A total of 36 polymorphisms (core chromosomal) occurred in predicted genes and separated historical and emergent *emm4* populations (Table S4). Importantly, several of the identified polymorphisms occurred in genes previously shown to alter virulence (Table 2). For example, all or nearly all emergent (SC3) strains contain inactivating mutations (historical strains with intact reading frames) in *ralp3* (98% [602/614]), which encodes a RofA family transcriptional regulator previously identified as important to GAS virulence (37), and *silA* (100% [614/614]), which encodes a response regulator of the Streptococcus invasion locus (*sil*) (38) (Table 2). Similarly, >86% of emergent strains contain a mutation predicted to result in a single amino acid change (T104I) in the regulator of proteinase RopB, an activator of the actively secreted virulence factor Streptococcus cysteine protease SpeB (39). We used casein hydrolysis (milk plate assays) to assess the functional impact of this polymorphism and found that the emergent strains produced a slightly larger clearance area relative to the historical strains consistent with increased SpeB production (Fig. S4A and B). Finally, 90% (377/417) of historical strains encode valine (V) at position 27 (a variably conserved region of the predicted DNA-binding domain [40]) in the multigene activator (Mga) protein (41). Conversely, only 22% (138/614) of emergent *emm4* strains possess the same valine, and these strains form a distinct subpopulation within SC3 (data not shown). The remaining emergent strains (476/614) have an Mga protein with alanine (A) at position 27 and thus have a V27A change relative to the historical strains.

**TABLE 2:** Select virulence genes/loci with changes differentiating emergent and historical clades

Virulence gene(s)	Type^*a*^	Locus^*b*^/description	No. of strains (%) with virulence gene	
			Emergent (SC3) (*n* = 614)	Historical (SC1) (*n* = 417)
*emm/enn* chimera	R	D1F63_08970; gene fusion between *emm* and *enn*	614 (100)	0 (0.0)
*ropB* T104I	SNP	D1F63_09065; single amino acid change (threonine to isoleucine) in regulator of SpeB, RopB	532 (86.6)	1 (0.2)
*silA*	Indel	D1F63_02180; single nucleotide insertion leading to frameshift in response regulator, SilA, of the Sil bacteriocin signaling system	614 (100)	29 (7.0)
*ralp3*	SNP	D1F63_03645; nonsense mutation in *ralp3* of the ERES pathogenicity island	602 (98.0)	2 (0.5)
*mga* A27V	SNP	D1F63_08980; single amino acid change (alanine to valine) in stand-alone regulator Mga	137 (22.5)	376 (87.0)

a Type of chromosomal change: nucleotide insertion/deletion (indel), recombination (R), single nucleotide polymorphism (SNP).

b Gene locus in the *emm4* GAS reference strain Duke (GenBank accession number CP031770).

10.1128/mSystems.00495-21.4 TABLE S4 Clade-defining core chromosomal differences between emergent and historical *emm4* populations. Download Table S4, XLSX file, 0.02 MB. Copyright © 2021 DebRoy et al. 2021 DebRoy et al. https://creativecommons.org/licenses/by/4.0/ This content is distributed under the terms of the Creative Commons Attribution 4.0 International license.

10.1128/mSystems.00495-21.10 FIG S4 *In vitro* growth characteristics and phenotypes following *in vivo* mouse and *ex vivo* human blood models of GAS disease in emergent and historical *emm4* GAS. (A and B) Casein hydrolysis (milk plate) assays of historical and emergent clade *emm4* GAS. The area of clearance was measured following growth on milk plates as described in Materials and Methods for historical and emergent clade strains as a group (A) and individually (B). Asterisk indicates *P* < 0.05 following Mann-Whitney U-test. (C and D) Growth of *emm4* GAS strains *in vitro* in nutrient-rich (THY, C) and glucose-limiting (C medium, D) conditions. Details of strain growth are provided in Materials and Methods. The dashed line indicates mid-exponential (ME) phase of growth. (E to G) Cumulative Kaplan-Meier survival curves of mice infected with four emergent (red) or four historical (blue) *emm4* GAS over a range of doses (CFUs). Groups of seven mice were used per strain per dose. (H) Bactericidal assay of emergent and historical clade strains. Error bars represent standard deviations following assays performed in quadruplicate using blood from two independent donors. Download FIG S4, JPG file, 1.0 MB. Copyright © 2021 DebRoy et al. 2021 DebRoy et al. https://creativecommons.org/licenses/by/4.0/ This content is distributed under the terms of the Creative Commons Attribution 4.0 International license.

We also identified a small number of locus variation between the clades representing ∼12 kb (<1%) of the core chromosome (data not shown). Most of these variations were in genes encoding surface proteins with known highly repetitive sequences and frequent strain-to-strain allelic variation such as the streptococcal collagen-like proteins (*sclA* and *sclB*) (42, 43), serum opacity factor (*sof*) (44), and fibronectin-binding protein (*fbpA*) (23). However, in contrast to single nucleotide changes and the *emm-enn* fusion, the observed gene variations were not clade defining and occurred in both emergent and historical strains.

### *emm4* clades exhibit significantly different gene variation in key transcriptional regulators.

**FIG 3:**
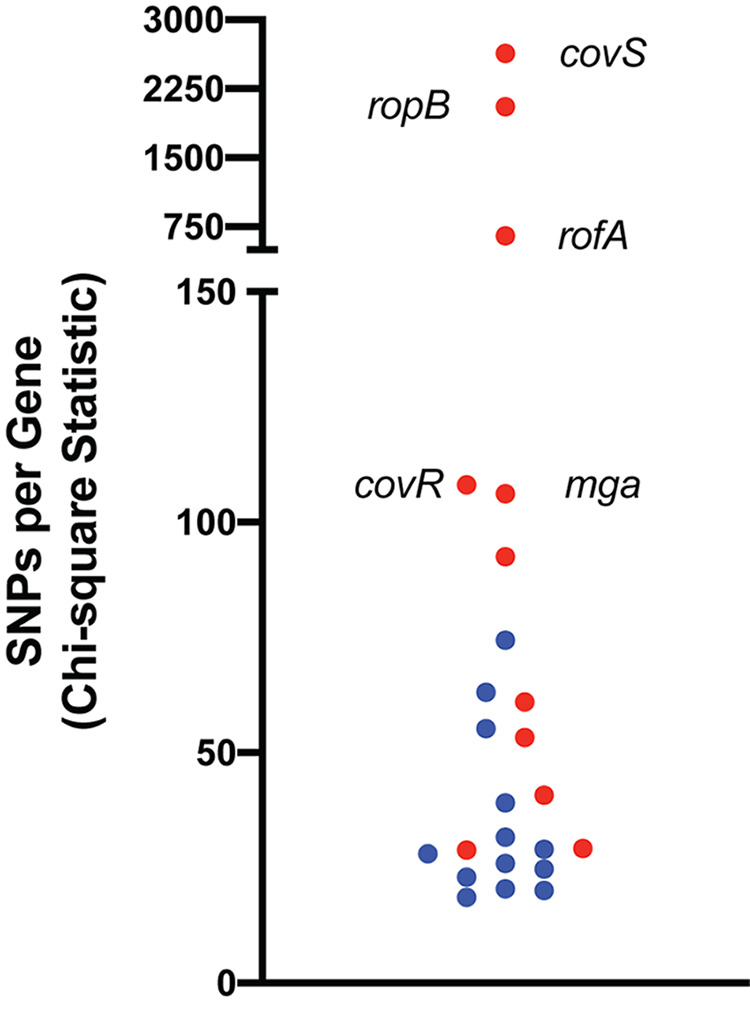
Highly polymorphic genetic loci identified within the *emm4* GAS population after exclusion of recombinant loci. Genes of known or predicted regulators (red) are indicated.

To gain insight into whether particular GAS loci were under selective pressure, we determined genetic variation at each of the 1,629 core genetic loci (relative to the reference strain Duke and excluding MGE) to define highly polymorphic genes. After exclusion of variation likely to have arisen due to repetitive elements or small recombination events, a total of 24 genes were identified as significantly highly polymorphic (Fig. 3 and Table S5). Interestingly, nearly half (10/24 [42%]) of the highly variant genes encoded known or predicted proteins of two-component systems (TCS) or stand-alone transcriptional regulators. Similar to previous investigations of GAS populations (5–7, 45), we identified the genes encoding the CovRS TCS as significantly highly polymorphic (Fig. 3). Mutations altering the activity of the CovRS TCS are well-known to aid in the transition from colonization to invasion (28, 46–49) and occur at relatively high frequencies in multiple GAS *emm* types (5, 6, 45, 50). In nearly all cases of regulator gene variation, observed mutations resulted in predicted modified proteins, with >25% of the mutations introducing a premature stop codon (131/505 [25.9%]) and nearly 70% leading to at least one amino acid change (350/505 [69.3%]) (Table S5).

10.1128/mSystems.00495-21.5 TABLE S5 List of highly polymorphic loci in the *emm4* GAS population, including clade-specific loci. Download Table S5, XLSX file, 0.01 MB. Copyright © 2021 DebRoy et al. 2021 DebRoy et al. https://creativecommons.org/licenses/by/4.0/ This content is distributed under the terms of the Creative Commons Attribution 4.0 International license.

To gain additional insights into mechanisms underlying the observed clonal emergence, we sought to determine whether the frequencies of gene variation differed between the historical and emergent clades. A total of 32 genes were identified in the *emm4* population with significantly higher variation levels compared to the rest of the genome of which 8 were also significantly highly polymorphic in both emergent and historical clade strains when analyzed separately (Table S5). Interestingly, excluding clade-defining mutations in *mga*, *ralp3*, and *ropB* (Table 2), we discovered that the proportion of strains with regulator-specific mutations differed significantly between clades (Table 3 and Table S5). Specifically, gene variation rates for *covS* (histidine kinase of CovRS TCS), *rofA*, and *ralp3* were significantly greater in historical clade strains compared to the emergent clade strains (Table 3). On the other hand, genetic variation in *mga* was observed to be significantly greater in emergent compared to historical strains (Table 3). The significantly different gene variation levels between the two *emm4* clades suggest different regulatory programs contributing to host-pathogen interaction.

**TABLE 3:** Frequency of mutation in known or predicted transcriptional regulators in *emm4* GAS population and by subclade

Gene	Locus tag^*a*^	Description^*b*^	Total *emm4* gene variation				Total *emm4*^*g*^ (*n* = 1,127)	SC3^*h*^ (*n* = 614)	SC1^*h*^ (*n* = 417)	*P* value^*i*^
			Mutations^*c*^	FS/NS/SYN^*d*^	Chi-square^*e*^	*P* value^*f*^				
*covS*	D1F63_01590	Sensor histidine kinase	157	48/106/3	2,635	0.0E+00	220 (19.5)	88 (14.4)	119 (28.5)	<0.001
*ropB* ^*j*^	D1F63_09080	Rgg family transcriptional regulator	103	17/86/0	2,056	0.0E+00	145 (12.9)	82 (13.4)	51 (12.2)	NS
*rofA*	D1F63_00785	HTH, Mga domain-containing protein	82	29/49/4	652	1.72E−140	137 (12.2)	55 (9.0)	73 (17.5)	<0.001
*mga* ^*k*^	D1F63_08980	Multiple gene activator, Mga	40	4/34/2	108	4.3E−22	114 (10.1)	95 (15.5)	*5 (1.2)*	<0.001
*covR*	D1F63_01585	DNA-binding response regulator	24	0/23/1	106	1.1E−21	28 (2.5)	10 (1.6)	16 (3.8)	NS
*fasA*	D1F63_01190	DNA-binding response regulator	20	7/13/0	61	9.6E−12	33 (2.9)	22 (3.5)	*8 (1.9)*	NS
*ralp3* ^*l*^	D1F63_03645	HTH, Mga domain-containing protein	30	11/12/7	53	4.8E−10	85 (7.5)	*13 (2.1)*	68 (16.3)	<0.001
*fasB* ^*m*^	D1F63_01180	Sensor histidine kinase	25	13/10/2	41	2.9E−7	118 (10.5)	57 (9.3)	*59 (14.1)*	NS
	D1F63_07540	HTH, Mga domain-containing protein	24	2/17/5	29	1.1E−4	38 (3.4)	*18 (2.9)*	*18 (4.3)*	NS

a Identifier in the reference genome Duke (CP031770).

b Putative function and/or conserved functional domains.

c Number of unique variations (mutations) in the given gene derived from the *emm4* population (*n* = 1,127).

d Number of mutations defined as frameshift or stop (FS), nonsynonymous (NS), or synonymous (SYN).

e Chi-square statistic based on total number of variations in the core genome (*n* = 10,594).

f *P* value based on chi-square test for the total *emm4* population following correction for multiple comparisons (Bonferroni).

g Number of strains in the *emm4* population (total *emm4*) with at least one of the defined gene mutations.

h Number of strains in emergent or historical clades (excluding ST38; *n* = 16) with at least one of the defined gene mutations. Italic values indicate that the indicated gene was not significantly highly polymorphic in the respective population (Table S5).

i *P* value (Fisher’s exact) comparing proportion of strains with at least one mutation in emergent versus historical clades following correction for multiple comparisons (Bonferroni).

j Excludes the clade-defining mutation T104I found in emergent/clade 2 strains.

k Excludes the clade-defining mutation A27V found in historical/clade 1 strains.

l Excludes the clade-defining nonsense mutation found in emergent/clade 2 strains and a synonymous SNP (*n* = 289 strains) in historical/clade 1 strains.

m Excludes a synonymous SNP (*n* = 492) occurring in both clades.

### Emergent *emm4* GAS strains exhibit a distinct transcriptome profile with decreased virulence gene expression.

**FIG 4:**
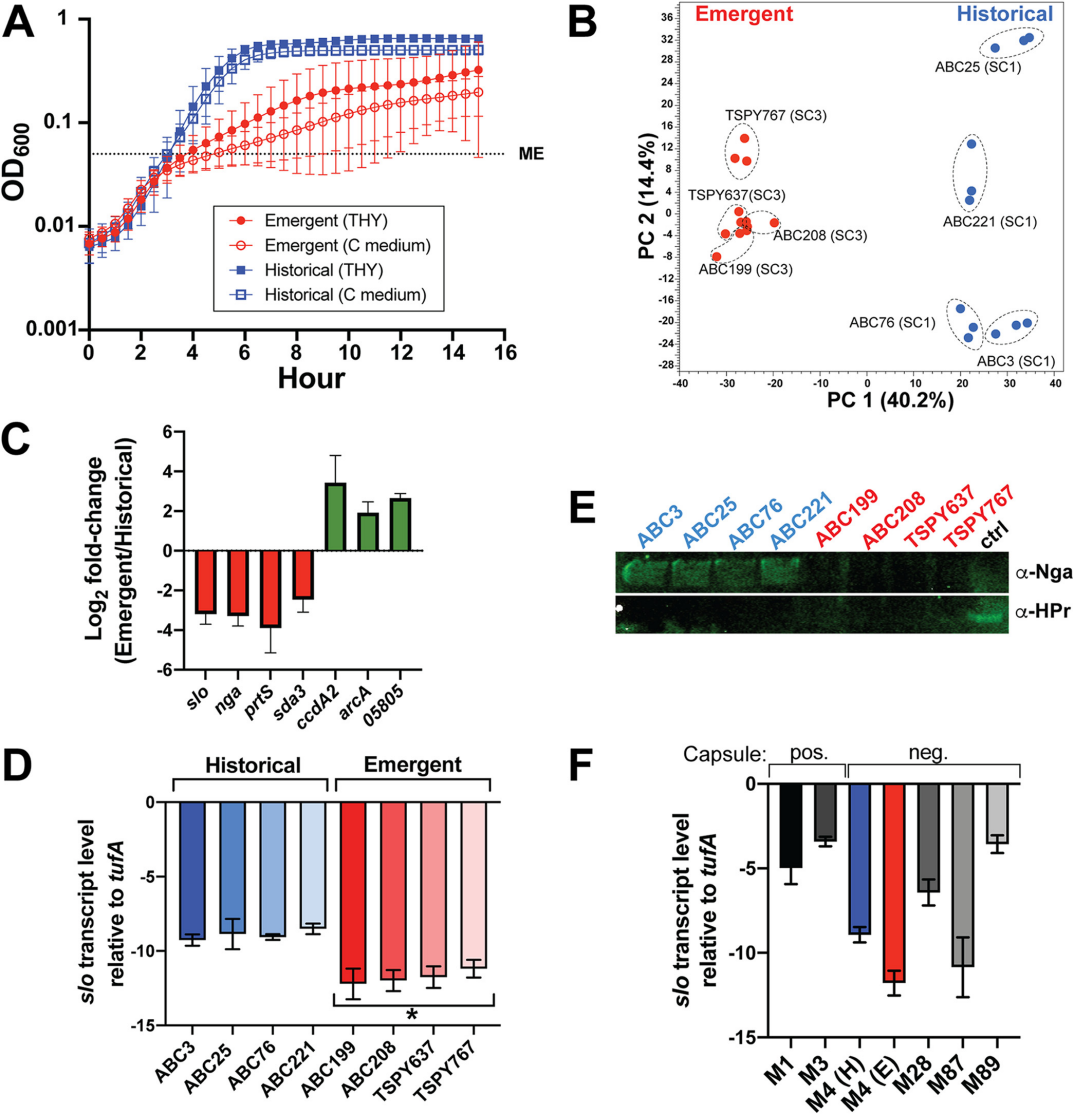
Emergent *emm4* GAS strains have altered *in vitro* growth and no increased SLO toxin production compared to historical strains. (A) Growth curves comparing historical (blue squares) to emergent (red circles) clade *emm4* GAS strains in nutrient-rich (THY; solid) or glucose-deficient (C medium; open) conditions. Error bars represent the standard deviations of four strains grown in biological triplicate. Dashed lines indicate mid-exponential (ME) phase of growth. (B) Principal-component analysis of RNA-seq comparing emergent (red) and historical (blue) clade strains. Individual strains with replicate samples are labeled and indicated using dashed circles. PC 1, principal component 1. (C) Log_2_ fold change in transcript levels derived from RNA-seq for selected genes derived from RNA-seq comparing four historical and four emergent *emm4* GAS strains. Error bars represent standard deviations. (D) Transcript levels (qRT-PCR) of *slo* from emergent or historical GAS cells grown in nutrient-rich media and harvested at ME as defined in panel A. The asterisk indicates a *P* value of <0.005 (unpaired *t* test). (E) Western blot analysis using anti-Nga antibody (α-Nga) of culture supernatants (SN) from historical (blue text) or emergent (red text) *emm4* GAS strains. Similar amounts of protein are demonstrated using anti-HPr. ctrl, control. (F) Transcript levels derived from qRT-PCR of *slo* from representative strains of *emm1* (MGAS2221), *emm3* (MGAS10870), *emm4*, *emm28* (strain TSPY902), *emm87* (strain TSPY1057), and *emm89* (strain MSPY1). For *emm4* GAS, transcript levels reflect four historical strains (in blue) and four emergent strains (in red). RNA was extracted from ME cultures as described in Methods. TaqMan qRT-PCR data for both panels D and F are means ± standard deviations of two biological replicates, with two technical replicates, done on two separate days. pos., positive; neg., negative.

The observed differences in regulator gene polymorphism frequency between the two clades, including regulators known to respond to metabolite availability, led us to hypothesize that emergent clade strains may differ from historical strains in *in vitro* growth characteristics and global gene regulation. For all subsequent clade comparisons, we identified four representative strains each from the SC1 (historical) and SC3 (emergent) clades (Table S3). All strains were wild type at key, known regulatory loci (e.g., TCS and stand-alone regulators) with the exception of clade-specific changes (Table 2 and Table S4). Thus, observed differences are likely to be representative of each clade. We first compared *in vitro* strain growth in nutrient-rich medium (THY) and glucose-limiting conditions (C medium). Overall, growth among emergent clade strains showed greater strain-to-strain variability compared to historical strains (Fig. 4A). Surprisingly, under both conditions, emergent clade strains had a marked growth defect beginning at approximately mid-exponential (ME) phase (Fig. 4A and Fig. S4C). The observed differences in growth were more pronounced under glucose-limiting conditions (C medium) (Fig. 4A and Fig. S4D), suggesting that emergent strains are sensitive to nutrient limitation. The lower optical densities (ODs) of the emergent strains correlated with lower CFU, indicating that the lower ODs were not due to an artifact (data not shown).

Next, we determined the *in vitro* transcriptomes of emergent and historical clade strains using RNA sequencing (RNA-seq) from cells grown in nutrient-rich medium and harvested at mid-exponential phase. These conditions were chosen given that growth differences were less pronounced in nutrient-rich media (Fig. 4A), and many GAS virulence factors are known to be maximally expressed during mid-exponential phase *in vitro* (26, 51). For RNA-seq analysis, the complete genome sequence was resolved for each of the eight strains and annotated using the prokaryotic genome annotation pipeline (PGAP) at NCBI (52). Via pangenome analysis, we identified 1,619 genes that were shared between the eight *emm4* genomes, and these were used to determine differential gene expression (DGE) between clades. Principal-component analysis demonstrated well-defined, clade-specific clustering of *emm4* strain transcriptomes (Fig. 4B). DGE was defined as an absolute fold difference of ≥1.5 and Bonferroni-corrected *P* value of <0.05. Excluding MGE, we found 62 genes upregulated and 141 genes downregulated in emergent strains relative to historical strains (Table S6). Highly upregulated genes in emergent clade strains involved the methionine oxidative response (*ccdA2*), arginine deiminase pathway (*arcA*), and genes encoding putative peptidoglycan-modifying enzymes (*05805*) (Fig. 4C). In striking contrast to previous GAS epidemic strain emergence investigations (6, 7, 53), we discovered that genes encoding the virulence factors *nga*, *slo*, *prtS* (interleukin 8 [IL-8] protease), and *sda3* (DNase) were among the most highly downregulated genes in emergent strains relative to historical strains (Fig. 4C). The observed differences in *nga* and *slo* transcript levels cannot be explained by sequence variation, as emergent and historical strains have identical *nga-slo* operons and promoter regions. Given that the T4 pilus was recently shown to contribute to colonization and immune evasion of *emm4* GAS (54), we also examined pilus gene transcription levels and again observed significantly decreased expression in emergent strains compared to historical clade strains (Table S6).

10.1128/mSystems.00495-21.6 TABLE S6 Differential gene expression determined by RNA sequencing in emergent versus historical *emm4* GAS strains. Download Table S6, XLSX file, 0.02 MB. Copyright © 2021 DebRoy et al. 2021 DebRoy et al. https://creativecommons.org/licenses/by/4.0/ This content is distributed under the terms of the Creative Commons Attribution 4.0 International license.

**FIG 5:**
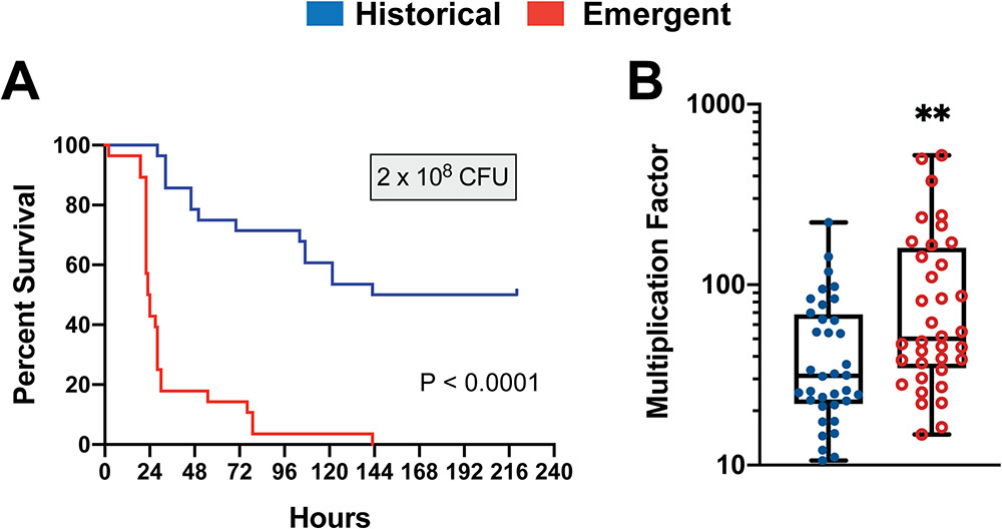
*In vivo* mouse and *ex vivo* human blood virulence assays comparing emergent and historical clade *emm4* GAS strains. (A) Cumulative Kaplan-Meier survival curves of mice (*n* = 7 per strains) infected with four historical (blue) or four emergent (red) *emm4* GAS strains (2 × 10^8^ CFU). The P value of  <0.0001 was determined by the log rank test. Survival curves following infection with other dose ranges are shown in Fig. S4 in the supplemental material. (B) Survival of emergent (red) or historical (blue) strains following exposure to human blood *ex vivo*. Error bars represent standard deviations of four emergent and four historical *emm4* GAS strains assayed in quadruplicate using three independent donors. The two asterisks indicate *P* < 0.05 (Mann-Whitney U test). Individual strain comparisons are shown in Fig. S4.

It is possible that the differences in growth between emergent and historical strains contributed to the observed *nga* and *slo* DGE. Given the pivotal role of Nga and SLO in previous GAS epidemic clonal emergence (6, 7, 53), we sought to confirm the lack of increased transcript levels in emergent clade strains. We isolated RNA from GAS strains grown in nutrient-rich medium at mid-exponential phase and assayed transcript levels of *slo* using quantitative reverse transcription-PCR (qRT-PCR). We found significantly reduced *slo* transcript levels in emergent strains compared to historical strains (Fig. 4D). Next, we sought to correlate Nga/SLO protein expression with transcript levels determined by qRT-PCR. Consistent with the decreased *nga* and *slo* transcript levels in the emergent strains, we observed less Nga in the supernatant derived from emergent strains (Fig. 4E). Inasmuch as acapsular strains (e.g., *emm4*) have been associated with high Nga/SLO toxin expression (25), we compared *nga* and *slo* transcript levels from historical and emergent *emm4* strains to strains representative of other GAS *emm* types with the high-level *nga* or *slo* promoter variant (25). We observed significantly lower *slo* transcript levels in *emm4* GAS compared to *emm1*, *emm3*, *emm28*, and *emm89* strains and similar levels compared to the *emm87* strain (Fig. 5F). Thus, in total, the above data suggest distinct regulatory programs governing emergent and historical *emm4* strains and that *emm4* clonal replacement did not result from augmented Nga/SLO toxin production.

### Emergent strains are more virulent compared to historical *emm4* GAS strains.

Emergence of novel GAS strains resulting in clonal replacement has previously been associated with enhanced virulence compared to historical strains (5–7). Given the nearly complete replacement observed in our temporal analysis and despite the seemingly paradoxical *in vitro* growth difference, we hypothesized that emergent *emm4* strains possess increased virulence compared to historical strains in models of GAS disease. The same historical and emergent strains used in transcriptomic analyses were used for virulence assays (Table S3). In a model of GAS bacteremia, groups of mice were inoculated with one of five doses of GAS (range, 5 × 10^6^ to 6 × 10^8^) in order to determine the dose at which 50% of the mice become moribund (LD_50_). Mice exhibited a dose response to GAS infection with either emergent or historical clade strains (Fig. S4E to S4G). With the exception of the lowest dose, mice infected with emergent clade GAS strains exhibited significantly decreased survival compared to historical *emm4* GAS strains. For example, the median survival time when infected with 2 × 10^8^ CFU (Fig. 5A) was 23.5 h in emergent strains compared to 181.5 h for historical strains. Next, we compared the ability of emergent and historical clade strains to survive in human blood *ex vivo* using the Lancefield bactericidal assay (55). In agreement with the results from the mouse challenge, emergent clade *emm4* strains demonstrated significantly increased growth in human blood compared to the historical strains (Fig. 5B), although there was substantial strain-to-strain variability (Fig. S4H). The increased virulence observed in emergent *emm4* GAS strains that have nearly replaced all previously circulating strains herein suggests that mechanisms that do not involve augmentation of Nga/Slo toxin expression can lead to GAS epidemic emergence.

## DISCUSSION

Whole-genome sequencing (WGS) of large cohorts of clinical isolates facilitated by the dramatic decrease in sequencing costs over the past 15 years have allowed for clear detection of clonal emergence and spread of numerous major human bacterial pathogens such as USA300 methicillin-resistant Staphylococcus aureus (1, 2) and lineage 11 Neisseria meningitidis (4). Similarly, WGS of distinct GAS *emm* types has identified clonal emergence and replacement events resulting in tens of thousands of life-threatening human infections (5–7, 45). We previously detected an unrecognized *emm-enn* fusion event in contemporary *emm4* GAS isolates (27). Herein, we used WGS to analyze >1,000 *emm4* GAS strains of diverse temporal and geographic origins to ascertain the extent and broader molecular context of the *emm-enn* gene chimera. Our results show that there has been nearly complete strain replacement among *emm4* GAS in North America over the past 20 years. Importantly, this strain replacement is not defined by acquisition of exogenous DNA which has previously been the molecular basis attributed to GAS clonal replacement (6, 9).

A critical strength of our study relative to other investigations of bacterial clonal emergence was that the replacement event occurred during a period of active surveillance (CDC active surveillance for GAS began in 1997) which facilitated collection of large numbers of both historical and emergent isolates. In turn, this meant that we could use large-scale phylogenomic analyses to accurately select representative strains from both the historical and emergent populations on which to perform comparative studies rather than having to rely on a small number of convenience samples for the historical isolates. Inasmuch as a single nucleotide polymorphism can markedly change GAS phenotype (9, 56), having robust sample collections for both the historical and emergent clades markedly increases the confidence that our findings are generally applicable to the populations being studied. Additionally, the large number of historical and emergent isolates facilitated intra-*emm* type comparison of relative rates of gene polymorphisms. Intriguingly, the hypovirulent historical isolates had higher rates of *covS* mutation, which we have recently shown increases *emm4* virulence (57). Conversely, the emergent strains had higher rates of *mga* variation, which we postulate may reflect the *in vitro* growth issues we identified in the emergent strains given the critical role of Mga in GAS metabolism (41).

Nearly 10 years ago, *emm4* and *emm22*, were the first identified acapsular *emm* types, a finding which was explained by the absence of the capsule-encoding *hasABC* operon (21). This discovery was surprising given that the GAS hyaluronic acid capsule has been considered critical to GAS pathogenesis for nearly a century (58). However, in the decade since the initial recognition of acapsular *emm4* and *emm22* GAS, we and others have described numerous other *emm* types either lacking or having abrogating mutations in the *hasABC* operon (22, 25). Indeed, acapsular GAS bacteria account for >30% of invasive GAS isolates as determined by active CDC surveillance (24). To date, the majority of acapsular GAS investigation has focused on *emm89*, as strains lacking the *hasABC* operon have recently emerged from and replaced their encapsulated precursors to cause epidemics in both the United States and Europe (7, 53). The “new” *emm89* strains have several regions of large-scale genetic recombination relative to replaced strains (26). The GAS *emm* types that are the source of these recombined areas have not been fully defined, but it is clear that the emergent *emm89* clone contains numerous pieces of exogenous DNA that likely have contributed to its global spread. In contrast, we found emergent *emm4* GAS are not distinguished from the replaced strains by acquisition of exogenous DNA despite our use of multiple, complementary methodologies. Thus, the emergent *emm4* GAS strains are not defined by the presence of a new virulence factor, which also stands in contrast to various successful GAS clones such as contemporary *emm3* strains that contain an actively secreted phospholipase (*sla*) (15). Some 50% of new *emm4* strains contain the DNase Sdn which was very rarely identified in historical strains (Fig. 2), but half of the new strains lack Sdn, meaning that Sdn acquisition cannot explain emergence of the new strains. Our findings of small changes in endogenous DNA content rather than acquisition of exogenous DNA defining an emerging GAS clade echo those recently described for an *emm1* clone in England causing scarlet fever (59). Thus, together with the recent *emm1* data, our *emm4* clonal emergence findings suggest that increased focus needs to be paid to understanding mechanisms by which alteration of endogenous DNA content impacts GAS pathophysiology.

The GAS recombination event that has been most studied thus far involves the *nga* or *slo* operon and results in augmented transcript levels of the gene encoding the streptolysin O toxin through acquisition of a “high-activity” promoter variant (9, 25). Clonal replacement and emergence of both *emm1* (6) and *emm89* (7, 53) GAS have been at least partially ascribed to this particular event. Indeed, all of the genes differentially transcribed between currently circulating and replaced *emm1* strains were located in the *nga* or *slo* recombination area (6). Additionally, acquisition of a high-activity *nga* or *slo* promoter by a variety of *emm* types through recombination has been specifically associated with acapsular GAS strains (25). In stark contrast, we found no difference between the historical and emergent *emm4* GAS strains in terms of *nga/slo* region genetic composition. Additionally, we identified that both historical and emergent *emm4* GAS strains produce significantly lower *slo* transcript levels relative to strains with an identical high-activity *nga* or *slo* promoter. We also found that similarly low *nga* and *slo* transcript levels were present in an acapsular *emm87* strain with a high-activity *nga* or *slo* promoter. Therefore, our data challenge the notion that the *nga* or *slo* region is the sole driver of acapsular GAS strain emergence, and they conclusively show that acapsular strains can cause serious infections without elevation of *slo* transcript levels.

Our analyses have identified several marked phenotypic and genotypic differences between the historical and emergent strains, which complicates the ability to definitively associate a single trait with *emm4* GAS clonal emergence. Surprisingly, the emergent strains had a clear growth defect relative to the historical strains. As noticeable as this defect is under *in vitro* conditions, the epidemiologic data clearly show that it does not affect the overall fitness of the organism in the human host, as demonstrated by the hundreds of invasive GAS infections caused by the emergent *emm4* clade. Similarly, our RNA-seq data show markedly lower transcript levels of numerous key virulence factor-encoding genes such as *prtS* and *sda3* in the emergent strains. In contrast to the RNA-seq data, however, the emergent strains were more virulent in an intraperitoneal mouse challenge model and an *ex vivo* human blood model, findings which are consistent with *emm1* (6) and *emm89* (7, 53) studies in which the expanding clones were hypervirulent relative to the replaced strains. However, increased virulence in emergent *emm4* strains was not associated with alterations in the *nga* or *slo* region as has been clearly demonstrated for *emm1* and *emm89* epidemic strains. We identified multiple small-scale genetic events separating the emergent and historical *emm4* strains, such as the *emm-enn* gene fusion and early stop codons in genes encoding two regulatory proteins previously identified as impacting GAS pathophysiology. Additionally, the emergent strains contained a single amino acid change in RopB and produced slightly more of the broad-spectrum, actively secreted protease SpeB relative to historic stains. None of the genetic changes that we identified in the emerging *emm4* strains were shared with the 27 SNPs that defined the new *emm1* English strains discussed above (59). Interestingly, our extensive phylogenetic analysis did not reveal a stepwise acquisition of the genetic differences separating the emergent and historical strains suggesting that a previously unrecognized clone containing all of the changes may have been introduced into the population. Alternatively, the “intermediate” strains may not have caused invasive infection such that they were not available for our analysis. Which of these factors or combination of factors accounts for observed phenotypic differences is an active area of further investigation in our laboratories.

Another key difference between our investigation and other studies of GAS strain emergence relates to total disease burden. For both *emm1* and *emm89* GAS, the emerging clones caused markedly more total infections relative to the precursor strains (6, 7, 53), findings which in turn spurred subsequent investigation. In contrast, our study of *emm4* GAS was driven by the WGS identification of the *emm-enn* gene fusion in a single strain (27). Indeed, total *emm4* invasive disease rates have not markedly changed in the United States over the past 20 years, possibly suggesting a defined ecologic niche for *emm4* organisms. Thus, our findings show that even when total GAS disease due to a particular *emm* type is relatively stable, there may be sizable variation in clonal distribution. To this end, the public availability of WGS from invasive GAS strains provided by the CDC is likely to assist with identification and characterization of heretofore unrecognized shifts in GAS epidemiology.

In conclusion, via WGS of >1,000 strains collected over a 20+ year period of active surveillance, we have discovered nearly complete replacement of previously circulating *emm4* GAS strains by a new clone in high-income settings. In contrast to prior GAS clonal replacement investigations, emergent *emm4* GAS strains are defined by small changes in endogenous DNA, rather than acquisition of exogenous DNA, and have low *slo* transcript levels. Yet, emergent *emm4* strains still have marked phenotypic variation relative to the replaced strains, indicating that much remains to be learned regarding the factors driving proliferation of GAS clones within the human population.

## MATERIALS AND METHODS

### GAS strains used in this study.

GAS strains are listed in Table S1 in the supplemental material. A total of 583 strains were obtained from national-level surveillance performed by the CDC Active Bacterial Core Surveillance (ABCs) beginning in 1997 (60, 61). Likewise, from a total of 812 strains, a representative subset of strains (*n* = 203) were selected from surveillance derived from the Toronto Invasive Bacterial Diseases Network and Alberta Provincial Laboratory for Public Health. All strains were grown using standard laboratory media as previously described (62). Genome sequence information from the remaining strains was obtained from the NCBI Sequence Read Archive (see “Data availability” below).

### Genomic DNA extraction, short- and long-read sequencing, and bioinformatic methods.

For all short-read, Illumina-based sequencing, high-quality genomic DNA was extracted from GAS cells grown overnight at 37°C and 5% CO_2_ on SBA using the Qiagen DNeasy kit modified for Gram-positive bacteria as previously described (63). Genomic DNA used in Oxford Nanopore long-read sequencing was extracted from cells using a cetyl trimethylammonium bromide (CTAB) protocol adapted for GAS to enrich for longer DNA fragments. The quantity and quality of DNA were measured using a nanodrop spectrophotometer (ThermoFisher) or Take3 microvolume plate reader (BioTek). DNA libraries for Illumina sequencing were generated using the Illumina Nextera Flex kit per the manufacturer’s instructions. Library quality was ensured using an Agilent TapeStation and sequenced using either an Illumina MiSeq or HiSeq4000 instrument. Long-read sequencing libraries were generated using the Oxford Nanopore Technologies Rapid Barcoding kit and GridION instrument per the manufacturer’s instructions.

Raw Illumina-based platform sequencing reads were processed for *de novo* assembly and polymorphism discovery as previously described (63). Briefly, raw reads (150-bp paired end [PE] for HiSeq4000 or 300-bp PE for MiSeq) underwent error correction and *de novo* assembly using SPAdes (v3.12.0) (64). Corrected reads were mapped to the reference M4 GAS genome Duke (GenBank accession number CP031770 [28]) using SMALT (https://www.sanger.ac.uk/science/tools/smalt-0). Polymorphisms were identified using freebayes (v1.2.0) (65) and filtered for quality (minimum positional depth of coverage of 15 and minimum polymorphism frequency of 0.75). For phylogenetic reconstruction, polymorphisms were filtered to exclude insertions/deletions (prephix.py; https://github.com/codinghedgehog), concatenated (phrecon.py; https://github.com/codinghedgehog), and the subsequent alignment of biallelic SNPs fed into RAxML (v8.2) (36, 66). ClonalFrameML (36) and gubbins (35) were used to identify and account for potential recombination/horizontal gene transfer. The final newick files were manipulated using CLC Genomic Workbench (v20) to incorporate associated strain metadata.

The complete genome sequence was resolved for a subset (*n* = 8) of *emm4* GAS strains (Table S3). Strains were chosen to represent major subclades and included strains wild type at all known two-component systems and stand-alone regulators except in cases where the clade or subclade was defined by such a mutation. Complete genome assembly was performed using both Illumina short-read and Oxford Nanopore GridION long-read sequences. Average depth of coverage for completed genomes exceeded 300-fold and was at least 50-fold for both short- and long-read sequences. Complete hybrid (short- and long-read) assemblies were obtained using Unicycler (v0.4.6) (67). Final circular chromosomes were annotated using PGAP at NCBI (52). A pangenome analysis was performed on strains used in RNA-sequencing experiments (*n* = 8, Table S3) using Roary (v3.12.0) (68) to define shared gene content.

Identification of MGE was performed using SPAdes-corrected short reads and SRST2 (v0.2.0) (69). MGE genotype was inferred based on the presence/absence of site-specific integrases or secreted virulence factors using a published database (45). Virulence factor content (e.g., exotoxin and secreted DNase) was assessed for consistency by blastn (70) comparison of *de novo* assemblies to a custom database containing all known exotoxin and secreted DNase alleles (71). In addition, completed genomes (*n* = 14) were manually inspected for the presence/absence of MGE, associated virulence genes, and sites of integration.

A complete pangenome analysis was performed on the *emm4* population (*n* = 1,127) using Roary (68). Individual annotated genomes for Roary pangenome analysis were generated using SPAdes *de novo* assembly followed by annotation with Prokka (v1.13.3) (72). Output from the pangenome analysis was subsequently fed into PANINI (https://panini.pathogen.watch) for identification of clusters using the Barnes-Hut t-SNE (33). Following clustering by PANINI, results were visualized using Microreact (https://microreact.org/) together with the maximum likelihood, recombination-corrected, phylogenetic tree and associated metadata.

### *In vitro* growth analysis of selected *emm4* GAS strains.

Growth curves for the four historical and four emergent clade strains were performed in THY or C medium as previously described (62). Briefly, overnight cultures grown at 37°C and 5% CO_2_ were used to inoculate (1/50 volume) prewarmed medium and distributed to a 96-well microtiter plate. Growth was monitored in a BioTek Synergy H1 plate reader, and readings were obtained every 30 min following shaking to redistribute cells in individual wells. Individual strains were assayed in technical quadruplicate and in biological triplicate on two independent days. Milk plate assays were performed in technical quadruplicate as previously described (39).

### RNA extraction, sequencing, and analysis.

High-quality RNA was extracted from GAS cells grown to mid-exponential phase (optical density at 600 nm [OD_600_] of 0.5) in THY at 37°C and 5% CO_2_ using the Qiagen RNeasy minikit as previously described (73). The quantity and quality of RNA were assessed using nanodrop (ThermoFisher) and by Agilent TapeStation. RNA-seq libraries were prepared using the Illumina ScriptSeq Complete Bacteria (v3) kit per the manufacturer’s instructions. Libraries were sequenced (150-bp PE) using an Illumina HiSeq4000 instrument. RNA-seq analysis was performed using CLC Genomics Workbench (v20). Raw reads were processed and mapped to the reference genome ABC25 (CP049696). Analysis was restricted to core genome content (*n* = 1,619 open reading frames/genes) based on the pangenome analysis for the completed genomes. Significant differential gene expression was defined as an absolute fold difference of >1.5 and Bonferroni-corrected *P* value of <0.05.

### Targeted gene transcript analysis.

For TaqMan real-time qRT-PCR, strains were grown in duplicate on two separate occasions to mid-exponential phase in THY as described above and processed as described previously (74). Primers and probes used targeted *slo* (forward, GACCTTTAAAGAGTTGCAACGAAAA; reverse, GACCATAAGCTACGTTACTCACAAAGA; probe, TGTCAGCAATGAAGCCCCGCC) with *tufA* (forward, CAATCGTCACTATGCGCACAT, reverse, GAGCGGCACCAGTGATCAT; probe, CTCCAGGACACGCGGACTACGTTAAAAA) as an internal control.

### SLO immunoblot assay.

GAS strains were grown to mid-exponential phase in 45 ml of THY as described above. Cultures were centrifuged to separate cells, supernatants were precipitated with acetone and resuspended in 10 mM Tris and 150 mM NaCl. Protein concentration was estimated by the Bradford method, and equal amounts of total protein were separated by sodium dodecyl sulfate-polyacrylamide gel electrophoresis (SDS-PAGE) and transferred. HPr and Nga were detected using a custom-made antibody (75) and a commercially available (Abcam) polyclonal antibody, respectively, and the Odyssey imaging system as described previously (51).

### Murine model of GAS bacteremia.

All mouse studies were performed under a protocol approved by the Institutional Animal Care and Use Committee at the MD Anderson Cancer Center. Four historic (ABC3, ABC25, ABC76, and ABC221) and four emergent (ABC199, ABC208, TSPY637, and TSPY767) strains were used. For the LD_50_ studies, seven female BALB/c mice per dose per strain (Harlan-Sprague-Dawley) were injected intraperitoneally with a range (5 × 10^6^ to 6 × 10^8^) of GAS CFU as described previously (57). Mice were monitored for near-mortality over 7 days, and survival was compared using Kaplan-Meier analysis. Differences in survival were calculated using a Mantel-Cox (log rank) analysis with a *P* value of <0.05 considered statistically significant.

### Lancefield assays.

Bactericidal assays were performed as previously described (56) under a protocol approved by the Committee for the Protection of Human Subjects at the University of Texas (UT) Health/McGovern Medical School. Blood samples from a minimum of three healthy, nonimmune, adult donors were used for each strain and performed in quadruplicate. Multiplication factors were calculated by dividing the number of CFU per milliliter after 3 h of incubation by the initial inoculum.

### Statistical analyses.

All statistical analyses were performed using GraphPad/Prism (v8). Statistical comparisons of continuous variables used Student’s *t* test (normally distributed) or Mann-Whitney U test (nonnormally distributed). Survival curves for mouse infection studies were compared using log rank test. *P* values less than 0.05 were considered significant for all tests.

### Data availability.

All raw sequence data generated in this study are available at NCBI under BioProject accession number PRJNA609136. Complete annotated genome assemblies are available at GenBank under accession numbers CP049697 (ABC3), CP049696 (ABC25), CP049695 (ABC76), CP049691 (ABC199), CP049690 (ABC208), CP049689 (ABC221), CP049686 (TSPY637), and CP055246 (TSPY767). Raw sequence data for *emm4* GAS strains sequenced by the CDC ABCs are available under BioProject PRJNA395240.

## References

[1] Challagundla L, Luo X, Tickler IA, Didelot X, Coleman DC, Shore AC, Coombs GW, Sordelli DO, Brown EL, Skov R, Larsen AR, Reyes J, Robledo IE, Vazquez GJ, Rivera R, Fey PD, Stevenson K, Wang SH, Kreiswirth BN, Mediavilla JR, Arias CA, Planet PJ, Nolan RL, Tenover FC, Goering RV, Robinson DA. 2018. Range expansion and the origin of USA300 North American epidemic methicillin-resistant Staphylococcus aureus. mBio 9:e02016-17. doi:10.1128/mBio.02016-17. PMC5750399 29295910

[2] Planet PJ, Diaz L, Kolokotronis SO, Narechania A, Reyes J, Xing G, Rincon S, Smith H, Panesso D, Ryan C, Smith DP, Guzman M, Zurita J, Sebra R, Deikus G, Nolan RL, Tenover FC, Weinstock GM, Robinson DA, Arias CA. 2015. Parallel epidemics of community-associated methicillin-resistant Staphylococcus aureus USA300 infection in North and South America. J Infect Dis 212:1874–1882. doi:10.1093/infdis/jiv320. 26048971 PMC4655856

[3] Croucher NJ, Harris SR, Fraser C, Quail MA, Burton J, van der Linden M, McGee L, von Gottberg A, Song JH, Ko KS, Pichon B, Baker S, Parry CM, Lambertsen LM, Shahinas D, Pillai DR, Mitchell TJ, Dougan G, Tomasz A, Klugman KP, Parkhill J, Hanage WP, Bentley SD. 2011. Rapid pneumococcal evolution in response to clinical interventions. Science 331:430–434. doi:10.1126/science.1198545. 21273480 PMC3648787

[4] Tzeng YL, Bazan JA, Turner AN, Wang X, Retchless AC, Read TD, Toh E, Nelson DE, Del Rio C, Stephens DS. 2017. Emergence of a new Neisseria meningitidis clonal complex 11 lineage 11.2 clade as an effective urogenital pathogen. Proc Natl Acad Sci USA 114:4237–4242. doi:10.1073/pnas.1620971114. 28373547 PMC5402416

[5] Beres SB, Carroll RK, Shea PR, Sitkiewicz I, Martinez-Gutierrez JC, Low DE, McGeer A, Willey BM, Green K, Tyrrell GJ, Goldman TD, Feldgarden M, Birren BW, Fofanov Y, Boos J, Wheaton WD, Honisch C, Musser JM. 2010. Molecular complexity of successive bacterial epidemics deconvoluted by comparative pathogenomics. Proc Natl Acad Sci USA 107:4371–4376. doi:10.1073/pnas.0911295107. 20142485 PMC2840111

[6] Nasser W, Beres SB, Olsen RJ, Dean MA, Rice KA, Long SW, Kristinsson KG, Gottfredsson M, Vuopio J, Raisanen K, Caugant DA, Steinbakk M, Low DE, McGeer A, Darenberg J, Henriques-Normark B, Van Beneden CA, Hoffmann S, Musser JM. 2014. Evolutionary pathway to increased virulence and epidemic group A *Streptococcus* disease derived from 3,615 genome sequences. Proc Natl Acad Sci USA 111:E1768–E1776. doi:10.1073/pnas.1403138111. 24733896 PMC4035937

[7] Zhu L, Olsen RJ, Nasser W, de la Riva Morales I, Musser JM. 2015. Trading capsule for increased cytotoxin production: contribution to virulence of a newly emerged clade of *emm89 Streptococcus pyogenes*. mBio 6:e01378-15. doi:10.1128/mBio.01378-15. 26443457 PMC4611041

[8] Beres SB, Richter EW, Nagiec MJ, Sumby P, Porcella SF, DeLeo FR, Musser JM. 2006. Molecular genetic anatomy of inter- and intraserotype variation in the human bacterial pathogen group A *Streptococcus*. Proc Natl Acad Sci USA 103:7059–7064. doi:10.1073/pnas.0510279103. 16636287 PMC1459018

[9] Zhu L, Olsen RJ, Nasser W, Beres SB, Vuopio J, Kristinsson KG, Gottfredsson M, Porter AR, DeLeo FR, Musser JM. 2015. A molecular trigger for intercontinental epidemics of group A Streptococcus. J Clin Invest 125:3545–3559. doi:10.1172/JCI82478. 26258415 PMC4588293

[10] Bricker AL, Cywes C, Ashbaugh CD, Wessels MR. 2002. NAD+-glycohydrolase acts as an intracellular toxin to enhance the extracellular survival of group A streptococci. Mol Microbiol 44:257–269. doi:10.1046/j.1365-2958.2002.02876.x. 11967084

[11] Bricker AL, Carey VJ, Wessels MR. 2005. Role of NADase in virulence in experimental invasive group A streptococcal infection. Infect Immun 73:6562–6566. doi:10.1128/IAI.73.10.6562-6566.2005. 16177331 PMC1230891

[12] Zhu L, Olsen RJ, Lee JD, Porter AR, DeLeo FR, Musser JM. 2017. Contribution of secreted NADase and streptolysin O to the pathogenesis of epidemic serotype M1 Streptococcus pyogenes infections. Am J Pathol 187:605–613. doi:10.1016/j.ajpath.2016.11.003. 28034602 PMC5397666

[13] Limbago B, Penumalli V, Weinrick B, Scott JR. 2000. Role of streptolysin O in a mouse model of invasive group A streptococcal disease. Infect Immun 68:6384–6390. doi:10.1128/.68.11.6384-6390.2000. 11035749 PMC97723

[14] Sumby P, Porcella SF, Madrigal AG, Barbian KD, Virtaneva K, Ricklefs SM, Sturdevant DE, Graham MR, Vuopio-Varkila J, Hoe NP, Musser JM. 2005. Evolutionary origin and emergence of a highly successful clone of serotype M1 group A *Streptococcus* involved multiple horizontal gene transfer events. J Infect Dis 192:771–782. doi:10.1086/432514. 16088826

[15] Beres SB, Sylva GL, Barbian KD, Lei B, Hoff JS, Mammarella ND, Liu MY, Smoot JC, Porcella SF, Parkins LD, Campbell DS, Smith TM, McCormick JK, Leung DY, Schlievert PM, Musser JM. 2002. Genome sequence of a serotype M3 strain of group A *Streptococcus*: phage-encoded toxins, the high-virulence phenotype, and clone emergence. Proc Natl Acad Sci USA 99:10078–10083. doi:10.1073/pnas.152298499. 12122206 PMC126627

[16] Wessels MR, Bronze MS. 1994. Critical role of the group A streptococcal capsule in pharyngeal colonization and infection in mice. Proc Natl Acad Sci USA 91:12238–12242. doi:10.1073/pnas.91.25.12238. 7991612 PMC45412

[17] Wessels MR, Goldberg JB, Moses AE, DiCesare TJ. 1994. Effects on virulence of mutations in a locus essential for hyaluronic acid capsule expression in group A streptococci. Infect Immun 62:433–441. doi:10.1128/iai.62.2.433-441.1994. 8300204 PMC186126

[18] Wessels MR, Moses AE, Goldberg JB, DiCesare TJ. 1991. Hyaluronic acid capsule is a virulence factor for mucoid group A streptococci. Proc Natl Acad Sci USA 88:8317–8321. doi:10.1073/pnas.88.19.8317. 1656437 PMC52499

[19] Foley MJ, Wood WB, Jr. 1959. Studies on the pathogenicity of group A streptococci. II. The antiphagocytic effects of the M protein and the capsular gel. J Exp Med 110:617–628. doi:10.1084/jem.110.4.617. 13823728 PMC2137000

[20] Dale JB, Washburn RG, Marques MB, Wessels MR. 1996. Hyaluronate capsule and surface M protein in resistance to opsonization of group A streptococci. Infect Immun 64:1495–1501. doi:10.1128/iai.64.5.1495-1501.1996. 8613352 PMC173953

[21] Flores AR, Jewell BE, Fittipaldi N, Beres SB, Musser JM. 2012. Human disease isolates of serotype M4 and M22 group A *Streptococcus* lack genes required for hyaluronic acid capsule biosynthesis. mBio 3:e00413-12. doi:10.1128/mBio.00413-12. 23131832 PMC3487777

[22] Flores AR, McNeil JC, Shah B, Van Beneden C, Shelburne SA, III. 2019. Capsule-negative emm types are an increasing cause of pediatric group A streptococcal infections at a large pediatric hospital in Texas. J Pediatric Infect Dis Soc 8:244–250. doi:10.1093/jpids/piy053. 30085121 PMC8938855

[23] Chochua S, Metcalf BJ, Li Z, Rivers J, Mathis S, Jackson D, Gertz RE, Jr, Srinivasan V, Lynfield R, Van Beneden C, McGee L, Beall B. 2017. Population and whole genome sequence based characterization of invasive group A streptococci recovered in the United States during 2015. mBio 8:e01422-17. doi:10.1128/mBio.01422-17. 28928212 PMC5605940

[24] Li Y, Rivers J, Mathis S, Li Z, Velusamy S, Nanduri SA, Van Beneden CA, Snippes-Vagnone P, Lynfield R, McGee L, Chochua S, Metcalf BJ, Beall B. 2020. Genomic surveillance of Streptococcus pyogenes strains causing invasive disease, United States, 2016–2017. Front Microbiol 11:1547. doi:10.3389/fmicb.2020.01547. 32849323 PMC7396493

[25] Turner CE, Holden MTG, Blane B, Horner C, Peacock SJ, Sriskandan S. 2019. The emergence of successful Streptococcus pyogenes lineages through convergent pathways of capsule loss and recombination directing high toxin expression. mBio 10:e02521-19. doi:10.1128/mBio.02521-19. PMC6904876 31822586

[26] Beres SB, Kachroo P, Nasser W, Olsen RJ, Zhu L, Flores AR, de la Riva I, Paez-Mayorga J, Jimenez FE, Cantu C, Vuopio J, Jalava J, Kristinsson KG, Gottfredsson M, Corander J, Fittipaldi N, Di Luca MC, Petrelli D, Vitali LA, Raiford A, Jenkins L, Musser JM. 2016. Transcriptome remodeling contributes to epidemic disease caused by the human pathogen *Streptococcus pyogenes*. mBio 7:e00403-16. doi:10.1128/mBio.00403-16. 27247229 PMC4895104

[27] DebRoy S, Li X, Kalia A, Galloway-Pena J, Shah BJ, Fowler VG, Flores AR, Shelburne SA. 2018. Identification of a chimeric emm gene and novel emm pattern in currently circulating strains of emm4 group A Streptococcus. Microb Genom 4:e000235. doi:10.1099/mgen.0.000235. PMC6321872 30412460

[28] Galloway-Pena J, Clement ME, Sharma Kuinkel BK, Ruffin F, Flores AR, Levinson H, Shelburne SA, Moore Z, Fowler VG, Jr. 2016. Application of whole-genome sequencing to an unusual outbreak of invasive group A streptococcal disease. Open Forum Infect Dis 3:ofw042. doi:10.1093/ofid/ofw042. 27006966 PMC4800461

[29] Tonkin-Hill G, Lees JA, Bentley SD, Frost SDW, Corander J. 2018. RhierBAPS: an R implementation of the population clustering algorithm hierBAPS. Wellcome Open Res 3:93. doi:10.12688/wellcomeopenres.14694.1. 30345380 PMC6178908

[30] Didelot X, Croucher NJ, Bentley SD, Harris SR, Wilson DJ. 2018. Bayesian inference of ancestral dates on bacterial phylogenetic trees. Nucleic Acids Res 46:e134. doi:10.1093/nar/gky783. 30184106 PMC6294524

[31] Duchene S, Holt KE, Weill FX, Le Hello S, Hawkey J, Edwards DJ, Fourment M, Holmes EC. 2016. Genome-scale rates of evolutionary change in bacteria. Microb Genom 2:e000094. doi:10.1099/mgen.0.000094. 28348834 PMC5320706

[32] Remmington A, Haywood S, Edgar J, Turner CE. 19 May 2020. Cryptic prophages within a Streptococcus pyogenes genotype emm 4 lineage. bioRxiv 10.1101/2020.05.19.103838. PMC8115907 33245690

[33] Abudahab K, Prada JM, Yang Z, Bentley SD, Croucher NJ, Corander J, Aanensen DM. 2019. PANINI: pangenome neighbour identification for bacterial populations. Microb Genom 5:e000220. doi:10.1099/mgen.0.000220. PMC6521588 30465642

[34] Woodbury RL, Klammer KA, Xiong Y, Bailiff T, Glennen A, Bartkus JM, Lynfield R, Van Beneden C, Beall BW, Active Bacterial Core Surveillance Team. 2008. Plasmid-borne erm(T) from invasive, macrolide-resistant Streptococcus pyogenes strains. Antimicrob Agents Chemother 52:1140–1143. doi:10.1128/AAC.01352-07. 18180360 PMC2258515

[35] Croucher NJ, Page AJ, Connor TR, Delaney AJ, Keane JA, Bentley SD, Parkhill J, Harris SR. 2015. Rapid phylogenetic analysis of large samples of recombinant bacterial whole genome sequences using Gubbins. Nucleic Acids Res 43:e15. doi:10.1093/nar/gku1196. 25414349 PMC4330336

[36] Didelot X, Wilson DJ. 2015. ClonalFrameML: efficient inference of recombination in whole bacterial genomes. PLoS Comput Biol 11:e1004041. doi:10.1371/journal.pcbi.1004041. 25675341 PMC4326465

[37] Kreikemeyer B, Nakata M, Koller T, Hildisch H, Kourakos V, Standar K, Kawabata S, Glocker MO, Podbielski A. 2007. The *Streptococcus pyogenes* serotype M49 Nra-Ralp3 transcriptional regulatory network and its control of virulence factor expression from the novel eno ralp3 epf sagA pathogenicity region. Infect Immun 75:5698–5710. doi:10.1128/IAI.00175-07. 17893125 PMC2168351

[38] Eran Y, Getter Y, Baruch M, Belotserkovsky I, Padalon G, Mishalian I, Podbielski A, Kreikemeyer B, Hanski E. 2007. Transcriptional regulation of the sil locus by the SilCR signalling peptide and its implications on group A streptococcus virulence. Mol Microbiol 63:1209–1222. doi:10.1111/j.1365-2958.2007.05581.x. 17238919

[39] Olsen RJ, Laucirica DR, Watkins ME, Feske ML, Garcia-Bustillos JR, Vu C, Cantu C, Shelburne SA, III, Fittipaldi N, Kumaraswami M, Shea PR, Flores AR, Beres SB, Lovgren M, Tyrrell GJ, Efstratiou A, Low DE, Van Beneden CA, Musser JM. 2012. Polymorphisms in regulator of protease B (RopB) alter disease phenotype and strain virulence of serotype M3 group A *Streptococcus*. J Infect Dis 205:1719–1729. doi:10.1093/infdis/jir825. 22262791 PMC4447876

[40] Vahling CM, McIver KS. 2006. Domains required for transcriptional activation show conservation in the mga family of virulence gene regulators. J Bacteriol 188:863–873. doi:10.1128/JB.188.3.863-873.2006. 16428389 PMC1347361

[41] Hondorp ER, McIver KS. 2007. The Mga virulence regulon: infection where the grass is greener. Mol Microbiol 66:1056–1065. doi:10.1111/j.1365-2958.2007.06006.x. 18001346

[42] Lukomski S, Nakashima K, Abdi I, Cipriano VJ, Ireland RM, Reid SD, Adams GG, Musser JM. 2000. Identification and characterization of the scl gene encoding a group A *Streptococcus* extracellular protein virulence factor with similarity to human collagen. Infect Immun 68:6542–6553. doi:10.1128/IAI.68.12.6542-6553.2000. 11083763 PMC97748

[43] Lukomski S, Nakashima K, Abdi I, Cipriano VJ, Shelvin BJ, Graviss EA, Musser JM. 2001. Identification and characterization of a second extracellular collagen-like protein made by group A *Streptococcus*: control of production at the level of translation. Infect Immun 69:1729–1738. doi:10.1128/IAI.69.3.1729-1738.2001. 11179350 PMC98079

[44] Courtney HS, Pownall HJ. 2010. The structure and function of serum opacity factor: a unique streptococcal virulence determinant that targets high-density lipoproteins. J Biomed Biotechnol 2010:956071. doi:10.1155/2010/956071. 20671930 PMC2910554

[45] Kachroo P, Eraso JM, Beres SB, Olsen RJ, Zhu L, Nasser W, Bernard PE, Cantu CC, Saavedra MO, Arredondo MJ, Strope B, Do H, Kumaraswami M, Vuopio J, Grondahl-Yli-Hannuksela K, Kristinsson KG, Gottfredsson M, Pesonen M, Pensar J, Davenport ER, Clark AG, Corander J, Caugant DA, Gaini S, Magnussen MD, Kubiak SL, Nguyen HAT, Long SW, Porter AR, DeLeo FR, Musser JM. 2019. Integrated analysis of population genomics, transcriptomics and virulence provides novel insights into Streptococcus pyogenes pathogenesis. Nat Genet 51:548–559. doi:10.1038/s41588-018-0343-1. 30778225 PMC8547240

[46] Flores AR, Luna RA, Runge JK, Shelburne SA, III, Baker CJ. 2017. Cluster of fatal group A streptococcal *emm87* infections in a single family: molecular basis for invasion and transmission. J Infect Dis 215:1648–1652. doi:10.1093/infdis/jix177. 28383686

[47] Flores AR, Sahasrabhojane P, Saldaña M, Galloway-Peña J, Olsen RJ, Musser JM, Shelburne SA. 2014. Molecular characterization of an invasive phenotype of group A *Streptococcus* arising during human infection using whole genome sequencing of multiple isolates from the same patient. J Infect Dis 209:1520–1523. doi:10.1093/infdis/jit674. 24307742 PMC3997578

[48] Masuno K, Okada R, Zhang Y, Isaka M, Tatsuno I, Shibata S, Hasegawa T. 2014. Simultaneous isolation of *emm89*-type *Streptococcus pyogenes* strains with a wild-type or mutated *covS* gene from a single streptococcal toxic shock syndrome patient. J Med Microbiol 63:504–507. doi:10.1099/jmm.0.070300-0. 24464696

[49] Sumby P, Whitney AR, Graviss EA, DeLeo FR, Musser JM. 2006. Genome-wide analysis of group A streptococci reveals a mutation that modulates global phenotype and disease specificity. PLoS Pathog 2:e5. doi:10.1371/journal.ppat.0020005. 16446783 PMC1354197

[50] Ikebe T, Ato M, Matsumura T, Hasegawa H, Sata T, Kobayashi K, Watanabe H. 2010. Highly frequent mutations in negative regulators of multiple virulence genes in group A streptococcal toxic shock syndrome isolates. PLoS Pathog 6:e1000832. doi:10.1371/journal.ppat.1000832. 20368967 PMC2848555

[51] Horstmann N, Sahasrabhojane P, Saldana M, Ajami NJ, Flores AR, Sumby P, Liu CG, Yao H, Su X, Thompson E, Shelburne SA. 2015. Characterization of the effect of the histidine kinase CovS on response regulator phosphorylation in group A Streptococcus. Infect Immun 83:1068–1077. doi:10.1128/IAI.02659-14. 25561708 PMC4333468

[52] Tatusova T, DiCuccio M, Badretdin A, Chetvernin V, Nawrocki EP, Zaslavsky L, Lomsadze A, Pruitt KD, Borodovsky M, Ostell J. 2016. NCBI prokaryotic genome annotation pipeline. Nucleic Acids Res 44:6614–6624. doi:10.1093/nar/gkw569. 27342282 PMC5001611

[53] Turner CE, Abbott J, Lamagni T, Holden MT, David S, Jones MD, Game L, Efstratiou A, Sriskandan S. 2015. Emergence of a new highly successful acapsular group A *Streptococcus* clade of genotype *emm89* in the United Kingdom. mBio 6:e00622-15. doi:10.1128/mBio.00622-15. 26173696 PMC4502227

[54] Chen YH, Li SH, Yang YC, Hsu SH, Nizet V, Chang YC. 2020. T4 pili promote colonization and immune evasion phenotypes of nonencapsulated M4 Streptococcus pyogenes. mBio 11:e01580-20. doi:10.1128/mBio.01580-20. 32694142 PMC7374061

[55] Lancefield RC. 1957. Differentiation of group A streptococci with a common R antigen into three serological types, with special reference to the bactericidal test. J Exp Med 106:525–544. doi:10.1084/jem.106.4.525. 13475611 PMC2136803

[56] Flores AR, Jewell BE, Yelamanchili D, Olsen RJ, Musser JM. 2015. A single amino acid replacement in the sensor kinase LiaS contributes to a carrier phenotype in group A Streptococcus. Infect Immun 83:4237–4246. doi:10.1128/IAI.00656-15. 26283331 PMC4598398

[57] Galloway-Pena J, DebRoy S, Brumlow C, Li X, Tran TT, Horstmann N, Yao H, Chen K, Wang F, Pan BF, Hawke DH, Thompson EJ, Arias CA, Fowler VG, Jr, Bhatti MM, Kalia A, Flores AR, Shelburne SA. 2018. Hypervirulent group A Streptococcus emergence in an acaspular background is associated with marked remodeling of the bacterial cell surface. PLoS One 13:e0207897. doi:10.1371/journal.pone.0207897. 30517150 PMC6281247

[58] Stollerman GH, Dale JB. 2008. The importance of the group A *Streptococcus* capsule in the pathogenesis of human infections: a historical perspective. Clin Infect Dis 46:1038–1045. doi:10.1086/529194. 18444821

[59] Lynskey NN, Jauneikaite E, Li HK, Zhi X, Turner CE, Mosavie M, Pearson M, Asai M, Lobkowicz L, Chow JY, Parkhill J, Lamagni T, Chalker VJ, Sriskandan S. 2019. Emergence of dominant toxigenic M1T1 Streptococcus pyogenes clone during increased scarlet fever activity in England: a population-based molecular epidemiological study. Lancet Infect Dis 19:1209–1218. doi:10.1016/S1473-3099(19)30446-3. 31519541 PMC6838661

[60] Nelson GE, Pondo T, Toews KA, Farley MM, Lindegren ML, Lynfield R, Aragon D, Zansky SM, Watt JP, Cieslak PR, Angeles K, Harrison LH, Petit S, Beall B, Van Beneden CA. 2016. Epidemiology of invasive group A streptococcal infections in the United States, 2005–2012. Clin Infect Dis 63:478–486. doi:10.1093/cid/ciw248. 27105747 PMC5776658

[61] O’Loughlin RE, Roberson A, Cieslak PR, Lynfield R, Gershman K, Craig A, Albanese BA, Farley MM, Barrett NL, Spina NL, Beall B, Harrison LH, Reingold A, Van Beneden C, Active Bacterial Core Surveillance Team. 2007. The epidemiology of invasive group A streptococcal infection and potential vaccine implications: United States, 2000–2004. Clin Infect Dis 45:853–862. doi:10.1086/521264. 17806049

[62] Gera K, McIver KS. 2013. Laboratory growth and maintenance of *Streptococcus pyogenes* (the group A *Streptococcus*, GAS). Curr Protoc Microbiol 30:9D.2.1–9D.2.13. doi:10.1002/9780471729259.mc09d02s30. PMC3920295 24510893

[63] Sanson MA, Macias OR, Shah BJ, Hanson B, Vega LA, Alamarat Z, Flores AR. 2019. Unexpected relationships between frequency of antimicrobial resistance, disease phenotype and emm type in group A Streptococcus. Microb Genom 5:e000316. doi:10.1099/mgen.0.000316. PMC6927302 31755853

[64] Bankevich A, Nurk S, Antipov D, Gurevich AA, Dvorkin M, Kulikov AS, Lesin VM, Nikolenko SI, Pham S, Prjibelski AD, Pyshkin AV, Sirotkin AV, Vyahhi N, Tesler G, Alekseyev MA, Pevzner PA. 2012. SPAdes: a new genome assembly algorithm and its applications to single-cell sequencing. J Comput Biol 19:455–477. doi:10.1089/cmb.2012.0021. 22506599 PMC3342519

[65] Garrison E, Marth G. 2012. Haplotype-based variant detection from short-read sequencing. arXiv:1207.3907 [q-bio.GN]

[66] Stamatakis A. 2014. RAxML version 8: a tool for phylogenetic analysis and post-analysis of large phylogenies. Bioinformatics 30:1312–1313. doi:10.1093/bioinformatics/btu033. 24451623 PMC3998144

[67] Wick RR, Judd LM, Gorrie CL, Holt KE. 2017. Unicycler: resolving bacterial genome assemblies from short and long sequencing reads. PLoS Comput Biol 13:e1005595. doi:10.1371/journal.pcbi.1005595. 28594827 PMC5481147

[68] Page AJ, Cummins CA, Hunt M, Wong VK, Reuter S, Holden MT, Fookes M, Falush D, Keane JA, Parkhill J. 2015. Roary: rapid large-scale prokaryote pan genome analysis. Bioinformatics 31:3691–3693. doi:10.1093/bioinformatics/btv421. 26198102 PMC4817141

[69] Inouye M, Dashnow H, Raven LA, Schultz MB, Pope BJ, Tomita T, Zobel J, Holt KE. 2014. SRST2: rapid genomic surveillance for public health and hospital microbiology labs. Genome Med 6:90. doi:10.1186/s13073-014-0090-6. 25422674 PMC4237778

[70] Altschul SF, Gish W, Miller W, Myers EW, Lipman DJ. 1990. Basic local alignment search tool. J Mol Biol 215:403–410. doi:10.1016/S0022-2836(05)80360-2. 2231712

[71] Deniskin R, Shah B, Munoz FM, Flores AR. 2019. Clinical manifestations and bacterial genomic analysis of group A Streptococcus strains that cause pediatric toxic shock syndrome. J Pediatric Infect Dis Soc 8:265–268. doi:10.1093/jpids/piy069. 30085250 PMC6601382

[72] Seemann T. 2014. Prokka: rapid prokaryotic genome annotation. Bioinformatics 30:2068–2069. doi:10.1093/bioinformatics/btu153. 24642063

[73] Sanson M, Flores AR. 2020. Group A Streptococcus transcriptome analysis. Methods Mol Biol 2136:113–133. doi:10.1007/978-1-0716-0467-0_8. 32430816

[74] Horstmann N, Saldana M, Sahasrabhojane P, Yao H, Su X, Thompson E, Koller A, Shelburne SA, III. 2014. Dual-site phosphorylation of the control of virulence regulator impacts group A streptococcal global gene expression and pathogenesis. PLoS Pathog 10:e1004088. doi:10.1371/journal.ppat.1004088. 24788524 PMC4006921

[75] DebRoy S, Aliaga-Tobar V, Galvez G, Arora S, Liang X, Horstmann N, Maracaja-Coutinho V, Latorre M, Hook M, Flores AR, Shelburne SA. 2021. Genome-wide analysis of in vivo CcpA binding with and without its key co-factor HPr in the major human pathogen group A Streptococcus. Mol Microbiol 115:1207–1228. doi:10.1111/mmi.14667. 33325565 PMC8359418

[76] Chalker V, Jironkin A, Coelho J, Al-Shahib A, Platt S, Kapatai G, Daniel R, Dhami C, Laranjeira M, Chambers T, Guy R, Lamagni T, Harrison T, Chand M, Johnson AP, Underwood A, Scarlet Fever Incident Management Team. 2017. Genome analysis following a national increase in scarlet fever in England 2014. BMC Genomics 18:224. doi:10.1186/s12864-017-3603-z. 28283023 PMC5345146

