# Supplementary figures and images for: Population Genomics of emm4 Group A Streptococcus Reveals Progressive Replacement with a Hypervirulent Clone in North America
Source: mSystems. 2021 Aug 10;6(4):e00495-21. doi: 10.1128/mSystems.00495-21 (PMC8409732; doi:10.1128/mSystems.00495-21)

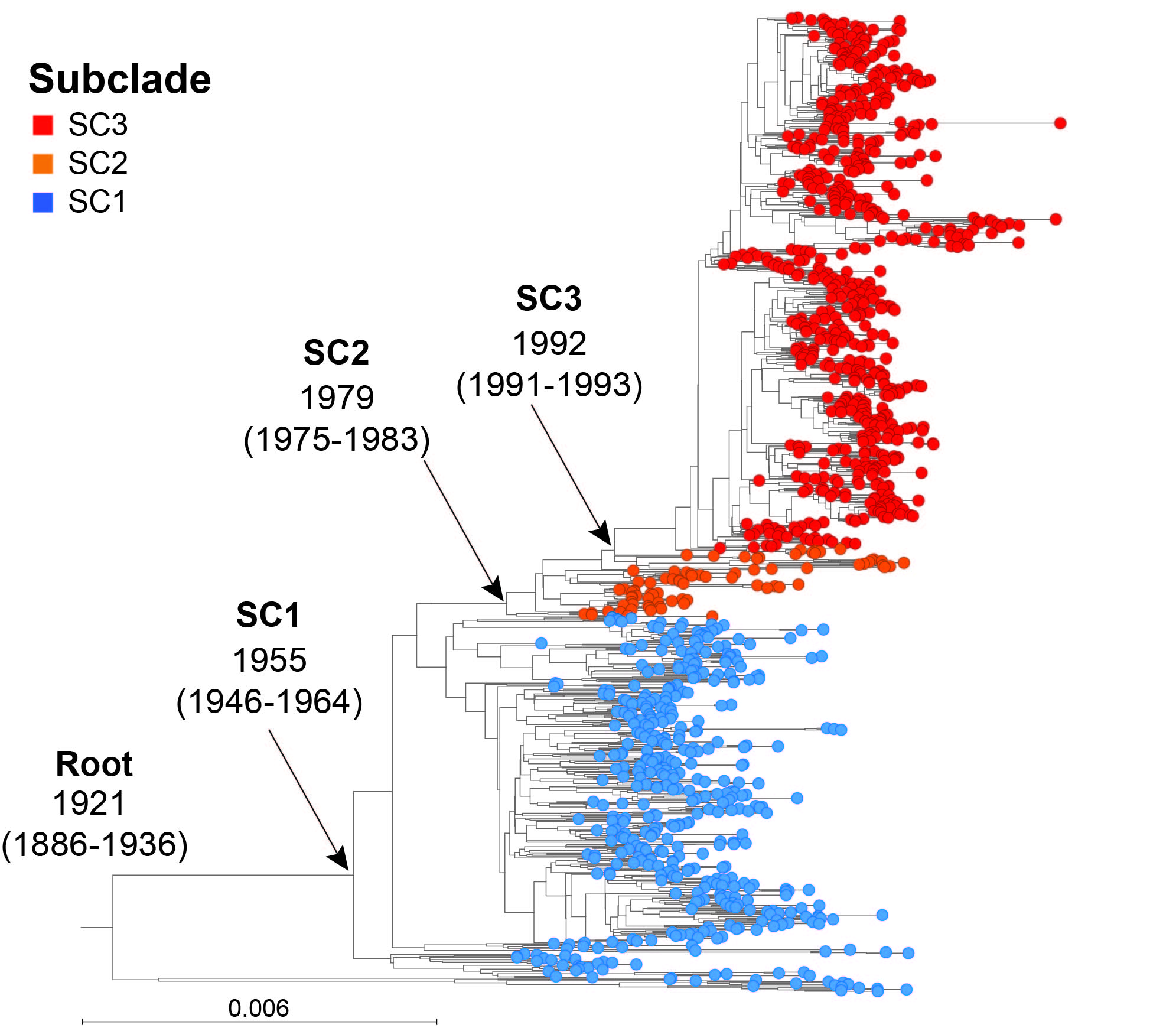

Supplement: FIG S1 [file msystems.00495-21-sf001.jpg]

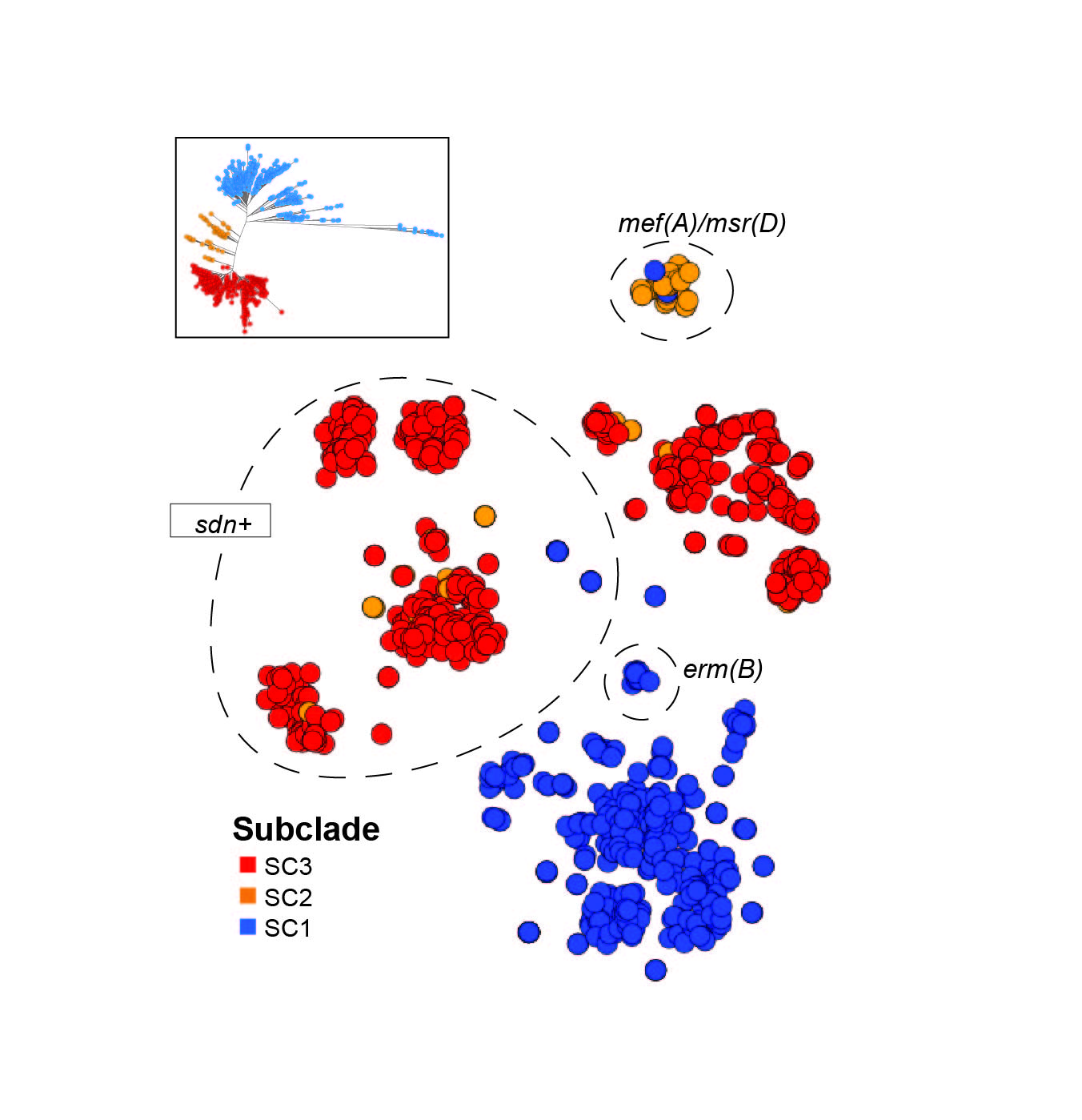

Supplement: FIG S2 [file msystems.00495-21-sf002.jpg]

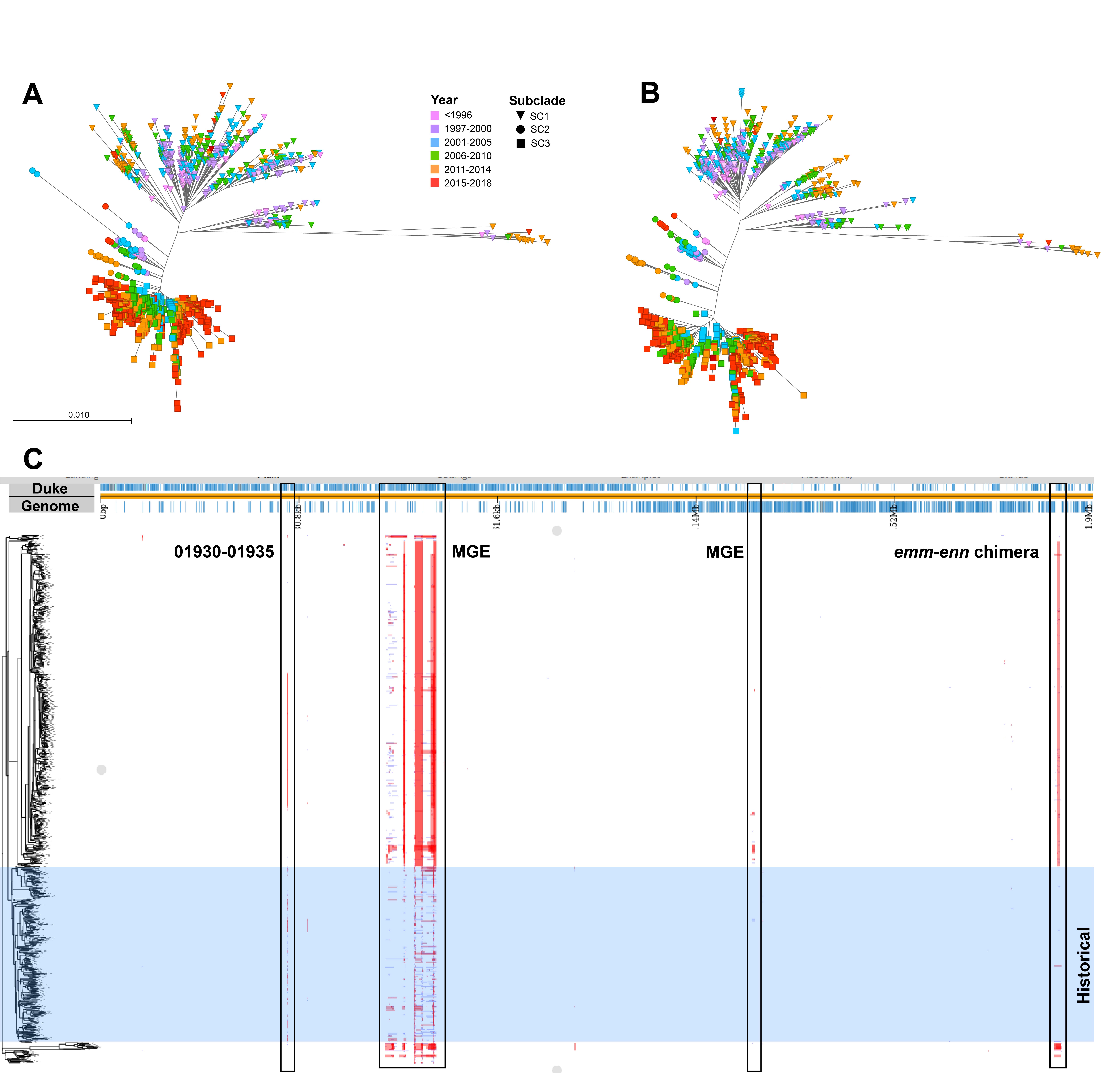

Supplement: FIG S3 [file msystems.00495-21-sf003.jpg]

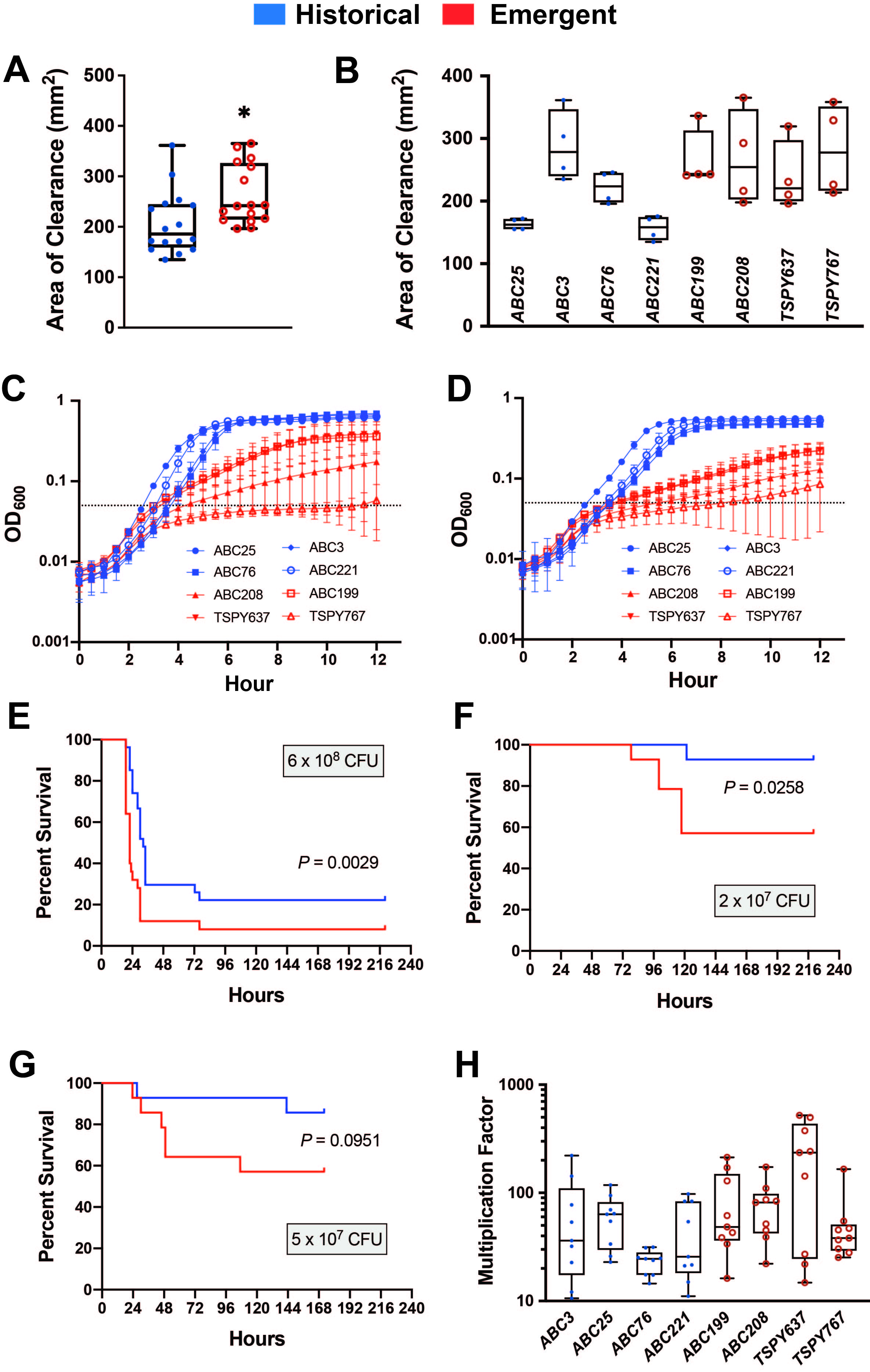

Supplement: FIG S4 [file msystems.00495-21-sf004.jpg]
