# Supplementary material for: Population Genomics of emm4 Group A Streptococcus Reveals Progressive Replacement with a Hypervirulent Clone in North America
Source: mSystems. 2021 Aug 10;6(4):e00495-21. doi: 10.1128/mSystems.00495-21 (PMC8409732; doi:10.1128/mSystems.00495-21)
Supplement: TABLE S2 [file msystems.00495-21-st002.docx]

**Supplemental Table S2**. Exotoxin and DNase content of *emm4* population

| **Genotype** | **SC1 (%) (n=433)** | **SC2 (%) (n=79)** | **SC3 (%) (n=614)** |
| --- | --- | --- | --- |
| *smeZ,speC, ssa* | 401 (92.6) | 78/79 (98.7) | 590 (96.1) |
| *spd1, spd3* | 399 (92.1) | 78/79 (98.7) | 591 (96.3) |
| *sdn* | 29 (6.7) | 27/79 (34.2) | 333 (54.2) |
