# Supplementary material for: Population Genomics of emm4 Group A Streptococcus Reveals Progressive Replacement with a Hypervirulent Clone in North America
Source: mSystems. 2021 Aug 10;6(4):e00495-21. doi: 10.1128/mSystems.00495-21 (PMC8409732; doi:10.1128/mSystems.00495-21)
Supplement: TABLE S3 [file msystems.00495-21-st003.docx]

**Supplemental Table S3.** Completed *emm4* GAS genomes used for transcriptome analysis.

| **Strain** | **SC** | **Acc. No.** | **Chr. (bp)** | **CDS** | **Pangenome** | |
| --- | --- | --- | --- | --- | --- | --- |
|  |  |  |  |  | Core (%) | Non (%) |
| ABC3 | 1 | CP049697 | 1,890,273 | 1908 | 84.9 | 289 (15.1) |
| ABC25 | 1 | CP049696 | 1,890,992 | 1909 | 84.8 | 290 (15.2) |
| ABC76 | 1 | CP049695 | 1,887,256 | 1902 | 85.1 | 283 (14.9) |
| ABC199 | 1 | CP049691 | 1,823,813 | 1804 | 89.7 | 185 (10.3) |
| ABC208 | 3 | CP049690 | 1,866,583 | 1861 | 87.0 | 242 (13.0) |
| ABC221 | 3 | CP049689 | 1,931,322 | 1970 | 82.2 | 351 (17.8) |
| TSPY637 | 3 | CP049686 | 1,823,857 | 1804 | 89.7 | 185 (10.3) |
| TSPY767 | 3 | CP055246 | 1,860,158 | 1847 | 87.7 | 228 (12.3) |
